# Cognitive function in patients with stable coronary heart disease: Related cerebrovascular and cardiovascular responses

**DOI:** 10.1371/journal.pone.0183791

**Published:** 2017-09-22

**Authors:** Mathieu Gayda, Vincent Gremeaux, Louis Bherer, Martin Juneau, Joffrey Drigny, Olivier Dupuy, Gabriel Lapierre, Véronique Labelle, Annik Fortier, Anil Nigam

**Affiliations:** 1 Cardiovascular Prevention and Rehabilitation Centre (ÉPIC), Montreal Heart Institute and University of Montreal, Montreal, Quebec, Canada; 2 Research Center, Montreal Heart Institute and University of Montreal, Montreal, Quebec, Canada; 3 Department of Medicine, Faculty of Medicine, University of Montreal, Montreal, Quebec, Canada; 4 INSERM - U1093 “Cognition, Action, et Plasticité Sensorimotrice”, Dijon, France; 5 PERFORM Centre, Department of Psychology, Concordia University, Montreal, Quebec, Canada; 6 Research Centre, Institut Universitaire de Gériatrie de Montreal, Montreal, Quebec, Canada; 7 Laboratory, MOVE (EA6314), Faculty of Sport Sciences, Université de Poitiers, Poitiers, France; 8 Montreal Health Innovations Coordinating Center, A Division of the Montreal Heart Institute, Montreal, Quebec, Canada; Kurume University School of Medicine, JAPAN

## Abstract

Chronic exercise has been shown to prevent or slow age-related decline in cognitive functions in otherwise healthy, asymptomatic individuals. We sought to assess cognitive function in a stable coronary heart disease (CHD) sample and its relationship to cerebral oxygenation-perfusion, cardiac hemodynamic responses, and $\dot{V}{\text{O}}_{2}$ peak compared to age-matched and young healthy control subjects. Twenty-two young healthy controls (YHC), 20 age-matched old healthy controls (OHC) and 25 patients with stable CHD were recruited. Cognitive function assessment included short term—working memory, perceptual abilities, processing speed, cognitive inhibition and flexibility and long-term verbal memory. Maximal cardiopulmonary function (gas exchange analysis), cardiac hemodynamic (impedance cardiography) and left frontal cerebral oxygenation-perfusion (near-infra red spectroscopy) were measured during and after a maximal incremental ergocycle test. Compared to OHC and CHD, YHC had higher $\dot{V}{\text{O}}_{2}$ peak, maximal cardiac index (CI max), cerebral oxygenation-perfusion (ΔO_2_ Hb, ΔtHb: exercise and recovery) and cognitive function (for all items) (P<0.05). Compared to OHC, CHD patients had lower $\dot{V}{\text{O}}_{2}$ peak, CI max, cerebral oxygenation-perfusion (during recovery) and short term—working memory, processing speed, cognitive inhibition and flexibility and long-term verbal memory (P<0.05). $\dot{V}{\text{O}}_{2}$ peak and CI max were related to exercise cerebral oxygenation-perfusion and cognitive function (P<0.005). Cerebral oxygenation-perfusion (exercise) was related to cognitive function (P<0.005). Stable CHD patients have a worse cognitive function, a similar cerebral oxygenation/perfusion during exercise but reduced one during recovery vs. their aged-matched healthy counterparts. In the all sample, cognitive functions correlated with $\dot{V}{\text{O}}_{2}$ peak, CI max and cerebral oxygenation-perfusion.

## Introduction

Cognitive impairment in patients with cardiovascular disease (CVD) includes deficits in memory, attention, executive function and psychomotor speed [1–4], and has been related to loss of gray matter in various brain regions [5], cerebral atrophy [6] and low resting cardiac output [7]. Whether vascular or blood flow abnormalities mediate some or all of these potential mechanisms remains a question of debate. Patients with CVD often exhibit lower cerebral oxygenation during maximal incremental exercise compared to healthy controls, cerebral oxygenation being related to $\dot{V}{\text{O}}_{2}$ peak, ventilatory threshold and resting cardiac function [8–10]. At the same time, chronic exercise has been shown to slow or prevent age-related decline in cognitive function among otherwise healthy individuals and has been related to improved cerebral blood flow [11]. In a recent study in both young and older healthy individuals, even acute exercise was associated with an improvement in cognitive function during exercise, indicating dynamic factors influence cognitive function [12]. Furthermore, at rest, higher cerebral blood flow (measured by transcranial Doppler) was associated with faster response times to cognitive test (Stroop) in both young and old and to a similar degree [12]. However, during exercise, cerebral blood flow (measured by transcranial Doppler) increased similarly in both groups and was unaltered by cognitive stroop tasks. In contrast, prefrontal cortical hemodynamic near-infra red spectroscopy (NIRS) measures [oxyhemoglobin (O_2_Hb) and total hemoglobin (tHb)] were differentially affected by exercise intensity, age and cognitive task; e.g., there were smaller increases in O_2_Hb and tHb in the older group between exercise intensities (P<0.05) [12]. These data indicate that: 1) Regardless of age, cognitive (executive) function is improved while exercising; 2) while cerebral blood flow (measured by transcranial Doppler) is strongly related to cognition at rest, this relation becomes uncoupled during exercise, and 3) there is dissociation between global CBF and NIRS regional cortical oxygenation and blood volume markers during exercise and engagement of prefrontal cognition.

Taken together, these previous studies suggest that several factors modulate cognitive function such as age, aerobic fitness and cardiovascular disease (such as coronary heart disease (CHD)) [11, 13, 14]. As well, based on the “vascular hypothesis” it has been suggested, particularly in patients with CHD, that a lower $\dot{V}{\text{O}}_{2}$ peak, cardiac function and cerebrovascular reserve (measured by NIRS) during exercise may be related to cognitive function [8–11, 13, 14], but this has not been explored yet. To our knowledge, no study has evaluated whether stable “fit” patients with CHD that regularly exercise (involved in long-term phase III cardiac rehabilitation) exhibit a similar degree of resting cognitive function compared to fit age-matched healthy controls, and whether cardiac and cerebral hemodynamic responses during maximal exercise may contribute to potential differences in cognitive function between groups. The objectives of this study were therefore 1) to evaluate cognitive function in a sample of fit CHD patients in comparison to a group of fit, age-matched healthy and younger controls, and 2) evaluate potential relationships between cognitive function, functional capacity and hemodynamic responses among groups. We hypothesized that cerebral oxygenation, cardiac hemodynamic responses, $\dot{V}{\text{O}}_{2}$ peak as well as cognitive function would be lower in patients with CHD compared to healthy controls, and that cognitive function would be related to cerebral oxygenation, cardiac hemodynamic responses and $\dot{V}{\text{O}}_{2}$ peakbased on the cerebrovascular reserve hypothesis [13].

## Material and methods

### Subjects

**Fig 1:**
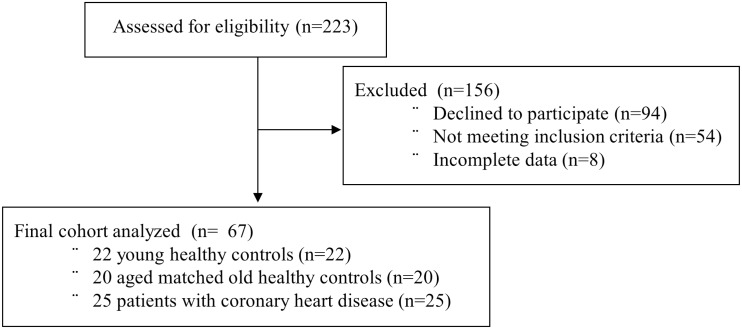
Flowchart of the inclusion of the subjects.

A total of 67 adults were prospectively enrolled from 2010 to 2014 in consecutive fashion, from the Cardiovascular Prevention and Rehabilitation Centre of the Montreal Heart Institute, including 25 fit patients with stable CHD, 20 age-matched healthy controls and 22 young healthy controls (Fig 1 Flow chart and the Inclusion/Exclusion Criteria section of the S1 File). Patients with CHD were defined as “stable” for their disease because of the following exclusion criteria used: recent acute coronary syndrome (<3 months), uncontrolled hypertension, recent bypass surgery intervention <3 months, recent percutaneous transluminal coronary angioplasty <6 months, left ventricular ejection fraction <45%, pacemaker or implantable cardioverter defibrillator, recent modification of medication <2 weeks. All subjects provided written informed consent and the protocol was approved by the Ethics Committee of the Montreal Heart Institute. The study was registered on ClinicalTrials.gov under identifier number: NCT03018561. All subjects underwent a baseline evaluation including a medical history, physical examination with measurement of height and weight, body composition (bioimpedance, Tanita, model BC418, Japan) and fasting blood sample (glucose and lipid profile) [15]. All subjects performed cognitive testing at rest and a maximal cardiopulmonary exercise test (CEPT) with gas exchange analysis. During CEPT, cerebral oxygenation and cardiac hemodynamic responses were measured continuously (see measurements section for details).

### Measurements

#### Cognitive function evaluation

Resting cognitive function was performed by a clinical neuropsychologist with training in cognitive test administration and evaluated using a validated paper-and-pencil neuropsychological battery test [16, 17]. Mini-Mental State examination (MMSE) and Geriatric Depression Scale (GDS) and were used to exclude patients suffering from depression and mental disease. A score < 26/30 on the MMSE and > than 10 on the GDS resulted in exclusion. This battery included the following tests: *a* Digit Span (Forward and Backward) (short-term and working memory). In this test, the neuropsychologist gave a series of numbers at the rate of about one per second. Following presentation, the subjects repeats the numbers in the order they were presented (Digits Forward) or in reverse order (Digits Backward). *b*. Digit Symbol Substitution Test (attention & processing speed), In this test, the participant had to associate symbols to numbers (1 to 9), in a table of numbers, by referring to a response key. The participant had 120 seconds to draw as many symbols as possible. *c*. Trail making test, part A and B. In the Trail Making Test part A, the participant had to connect numbers (from 1 to 25) with straight lines as fast as possible. In Part B, which measures flexibility, the participant had to alternate between letters in alphabetical order and numbers in ascending order (1-A-2-B-3-C, etc.) as fast as possible. *d*. D-KEFS Color-Word Interference Stroop Test: The Modified Stroop color test includes four conditions and provides a measure of inhibition and mental flexibility. In the reading condition (1), the participant had to read aloud color words as fast as possible. In the naming condition (2) they had to name the color of rectangles. In the inhibition condition (3), color-words were printed in a color that differed from their meaning (e.g., red printed in green) and the task was to name the ink color (green) and avoid reading the word. In the flexibility condition (4), the participant had to alternate between naming the color of the color-words, and reading the words (when the color-words appear in a square). Typically, scores in the more difficult conditions (3 & 4) are considered representative of executive control. In all conditions, word lists were printed on a sheet of paper and participants had to provide their answer verbally as fast as possible. e. Rey Auditory Verbal Learning Test (RAVLT, long-term verbal memory). In the RAVLT, participant must learn and remember a list of 15 words immediately after learning them and after a delay.

#### Maximal cardiopulmonary exercise testing

Exercise testing was performed on an ergocycle (Ergoline 800S, Bitz, Germany) with an individualized protocol that included a 3-min warm up at 20 Watts, followed by a power increase of 10 to 20 Watts/min until exhaustion at a free pedaling speed > 60 rpm [18–20]. Gas exchange was measured continuously at rest, during exercise, and after exercise cessation using a metabolic system (Oxycon Pro, Jaegger, Germany). The calibration of the flow module was accomplished by introducing a calibrated volume of air at several flow rates with a 3-liter pump. Gas analyzers were calibrated before each test using a standard certified commercial gas preparation (O_2_: 16%; CO_2_: 5%). Data were measured every four respiratory cycles during testing and then were averaged every 15 sec for minute ventilation ($\dot{V}\text{E}$, in l/min, BTPS), oxygen uptake ($\dot{V}{\text{O}}_{2}$, in l/min, STPD), carbon dioxide production ($\dot{V}{\text{CO}}_{2}$, in l/min, STPD) [18, 21]. Maximal exercise test lasted until the attainment of one of the two primary maximal criteria: (A) a plateau of $\dot{V}{\text{O}}_{2}$ despite an increase in cadence, (B) R.E.R > 1.1, or one of the two secondary maximal criteria: (C) measured maximal heart rate attaining 95% of age-predicted maximal heart rate, (D) inability to maintain the cycling cadence, (E) subject exhaustion with cessation caused by fatigue and/or other clinical symptoms (dyspnea, abnormal BP responses) or ECG abnormalities that required exercise cessation. The ventilatory threshold was determined using a combination of the V-slope, ventilatory equivalents, and end-tidal oxygen pressure methods. The highest $\dot{V}{\text{O}}_{2}$ value reached during the exercise phase of each test was considered as the $\dot{V}{\text{O}}_{2}$ peak and peak power output (PPO) was defined as the power output reached at the last fully completed stage [18–20].

#### Cerebral oxygenation/perfusion

Cerebral oxygenation/perfusion was measured using a near-infrared spectroscopy (NIRS) system (Oxymon Mk III, Artinis Medical, Netherlands) during maximal exercise and recovery [19, 22, 23]. Optodes were placed on the left prefrontal cortical area between Fp1 and Fp3, according the modified international EEG 10–20 system [19, 22, 23]. During exercise test, optodes were secured with a tensor bandage wrapped around the forehead, a neoprene pad was place between the skin and the optodes plastic holder and ambient room light (dimmer) was reduced. To correct for scattering of photons in the tissue, a differential path-length factor of 5.93 was used for the calculation of absolute concentration changes with an interoptode distance of 45 mm. Data were sampled at 10 Hz during the rest period (3 min), the exercise phase and the 5-min recovery period. Data were displayed in real time and stored on disk for off-line analysis. Raw NIRS signals were filtered via the oxysoft/DAQ software (Artinis Medical, Netherlands) using a running average function with a filter width of 1. Thereafter, NIRS signals were exported into excel files with the oxysoft/DAQ software at 0.2 Hz for statistical treatment. Relative concentration changes (ΔμM) were measured from resting baseline of oxyhaemoglobin (ΔO_2_Hb), deoxyhaemoglobin (ΔHHb), total haemoglobin (ΔtHb) = (ΔO_2_Hb)+ (HHb) and differential haemoglobin (ΔHb diff.) = (ΔO_2_Hb)-(HHb). The baseline period was set at the end of the 3-min resting period, defined as 0 μM [19, 22, 23].

#### Cardiac hemodynamics

Cardiac hemodynamics were measured continuously at rest, during exercise and recovery using an impedance cardiography device (PhysioFlow^®^, Enduro model, Manatec, France) as previously published [19, 20, 23]. This noninvasive technique was previously found to be valid, accurate, and reproducible at rest and during exercise in healthy subjects and CHD patients [24–29]. Data were averaged every 15 consecutive heartbeats for cardiac index (CI: in l/min^/^m^2^, stroke volume index (SVi: in ml/m^2^), heart rate (in beats/min), end-diastolic and end-systolic volume index (EDVi and ESVi: in ml/m^2^), left cardiac work index (LCWi: in kg.m/m^2^), left ventricular ejection fraction (in %) and systemic vascular resistance index (SVRi: in dynes/s/cm^5^/m^2^).

### Statistical analysis

Results are presented as mean ± standard deviation except where otherwise indicated. Statistical analysis was performed using Statview software 5.0 and SAS version 9.4 (SAS, Cary, USA). Normal Gaussian distribution of the data was verified by the Shapiro–Wilk test. A one-way ANOVA (groups) was used to compare cardiopulmonary, hemodynamic and cognitive function variables. A two-way ANOVA (groups x time) was performed to compare brain NIRS parameters data during exercise and recovery between healthy controls and CHD patients. A Bonferroni post-hoc test was used to localize differences. Statistical significance was set at P<0.05 level for all analysis. Relationships between $\dot{V}{\text{O}}_{2}$ peak, CI max, NIRS variables and cognitive function items were performed using a Pearson coefficient of correlation (R) and statistical significance was corrected according to the number of correlations and set at P<0.005. Composite scores were calculated form the neuropsychological battery that assessed four cognitive domains: memory, speed of processing, executive functioning and verbal memory. All cognitive scores were first transformed in standardized z-scores and then averaged to provide a composite score for each domain. The composite z score were calculated as follow: 1) working memory = (forward + backward z scores)/2, 2) Speed of processing = (DSST+Trail A+Stroop 1+Stroop 2 z scores)/4, 3) Executive functioning = (Trail B+Stroop 3+Stroop 4 z scores)/3, 4) Verbal memory = (Immediate recall + delayed recall + Recognition + A1-15 z scores)/4. We sought to identify predictors of each of these four composite z score. Univariate analysis using Spearman correlation coefficient (to account for light deviation from normal distribution) was first performed to highlight potential predictors. All variables that showed a p≤0.05 in the univariate analysis were included in a stepwise linear regression analysis to identify independent predictors of each composite z score. Despite the light deviation from normal distribution for some predictors, the analysis of the residuals for each model shows that all underlying assumptions of linear regression were respected without the need to use data transformation. For a multiple linear regression model on predicting cognitive function which already includes 5 covariates with a squared multiple correlation R^2^ of 0.50, a sample size of 80 will have 80% power to detect at alpha = 0.05 an increase in R^2^ of 0.046 due to including the factor of interest (ex: ΔO_2_Hb). The number of covariates (ex: from 1 to 15) has only a very small effect on the results.

## Results

### Clinical characteristics

Baseline characteristics of all three groups are outlined in Table 1. The prevalence of traditional risk factors was significantly higher in patients with CHD relative to age-matched controls, both of these groups being significantly older than young healthy controls (See the Results section on Clinical characteristics of the S1 File for details).

**Table 1:** Clinical characteristics in young, old healthy controls and CHD patients.

	Young healthy controls (*n* = 22)	Old healthy controls (*n* = 20)	Patients with CHD (*n* = 25)
Age (years)	33 ± 11 ^a^^,^^b^ ^§^	67 ± 6	70 ± 8
Height (cm)	173 ± 6	170 ± 8	170 ± 8
Female sex	7 (31%)	4 (20%)	2 (8%)
Smoking	0 (0%)	0 (0%)	0 (0%)
Hypertension ^1^	0 (0%)	0 (0%)	15 (60%)
Diabetes ^2^	0 (0%)	0 (0%)	7 (28%)
History of dyslipidemia	0 (0%)	2 (10%)	20 (80%)
Obesity ^3^	0 (0%)	0 (0%)	11 (44%)
Prior MI	0 (0%)	0 (0%)	10 (40%)
Prior PCI	0 (0%)	0 (0%)	9 (36%)
Prior CABG	0 (0%)	0 (0%)	8 (32%)
**Medication**			
Beta–blockers	0 (0%)	0 (0%)	14 (58%)
ACE inhibitors	0 (0%)	0 (0%)	8 (32%)
Antiplatelet agents	0 (0%)	0 (0%)	24 (96%)
Angiotensin receptor blockers	0 (0%)	0 (0%)	6 (24%)
Statin	0 (0%)	1 (5%)	23 (92%)
Calcium channel blockers	0 (0%)	1 (5%)	9 (36%)
Nitrates	0 (0%)	0 (0%)	3 (12%)
Hypoglycemic agents	0 (0%)	0 (0%)	3 (12%)
**Body composition**			
Body mass (kg)	69 ± 8 ^b^*	70 ± 8 ^c^*	77 ± 11
BMI (kg/m^2^)	23 ± 2 ^b^^§^	24 ± 1 ^c^^†^	26 ± 2
Waist circumference (cm)	83 ± 7 ^b^^§^	89 ± 7 ^c^^†^	97 ± 9
Lean body mass (kg)	57 ± 8	55 ± 9	57 ± 8
FM percentage (%)	18 ± 7 ^a^*^,^ ^b^^§^	22 ± 4 ^c^*	26 ± 5
Trunk FM percentage (%)	16 ± 7 ^a^^†^^,^ ^b^^§^	22 ± 4 ^c^^§^	28 ± 5

^1^ Rest SBP ≥ 130 mmHg;

^2^ glucose ≥ 7 mmol/l;

^3^ BMI > 30 kg/m^2^

Group effect:

^a^ = young vs. older,

^b^ = young vs. CHD patients,

^c^ = older vs. CHD patients,

* = P<0.05,

^†^ = P<0.01,

^§^ = P<0.0001.

ACE, angiotensin–converting enzyme; CABG, coronary artery bypass grafting surgery; CHD, coronary heart disease; MI, myocardial infarction; PCI, percutaneous coronary intervention. BMI: body mass index, FM: fat mass, SBP: systolic blood pressure, DBP: diastolic blood pressure.

### Cognitive function parameters

Table 2 describes cognitive function parameters in all three groups. Briefly, except for GDS which was similar between groups, young healthy control subjects scored significantly higher on all tasks followed by age-matched controls then CHD patients. (See the Results section on Cognitive function parameters of the S1 File for details).

**Table 2:** Cognitive function parameters in young, old healthy controls and CHD patients.

	Young healthy controls (*n* = 22)	Old healthy controls (*n* = 20)	Patients with CHD (*n* = 25)	ANOVA P value
**Education (years)**	17 ± 1 ^a^*^,^^b^*	14 ± 3	15 ± 3	0.0277
**Mental disease / depression symptomatology**				
GDS (/30)	1.7 ± 1.3	1.8 ± 1.9	3.1 ± 3.2	0.2072
MMSE (/30)	29.2 ± 0.8 ^b^^†^	28.7 ± 1.1	28.1 ± 1.1	0.0107
**Short term and working memory**				
Forward Span	11.9 ± 1.7 ^a^^‡^^,^^b^^§^	9.9 ± 1.3 ^c^^†^	8.3 ± 1.7	<0.0001
Backward Span	8.6 ± 2.7 ^b^^§^	7.4 ± 2.0 ^c^^†^	5.7 ± 1.6	<0.0001
**Perceptual abilities and processing speed**				
DSST	88.7 ± 12.0 ^a^^§^^,^^b^^§^	65.8 ± 13.2 ^c^^†^	55.1 ± 13.8	<0.0001
Trail A (s)	23.35 ± 7.99 ^a^^§^^,^^b^^§^	40.80 ± 10.93	43.93 ± 12.65	<0.0001
Stroop 1(s)	24.05 ± 3.90 ^a^^†^^,^^b^^§^	31.60 ± 7.82	32.82 ± 7.28	0.0003
Stroop 2 (s)	17.81 ± 2.29 ^a^^†^^,^^b^^†^	23.74 ± 8.82	22.31 ± 3.29	0.0043
**Cognitive inhibition and flexibility**				
Trail B (s)	48.99 ± 13.99 ^a^^†^^,^^b^^§^	77.62 ± 20.32 ^c^^†^	112.24 ± 47.26	<0.0001
Stroop 3 (s)	38.66 ± 11.41 ^a^^‡^^,^^b^^§^	55.38 ± 12.79 ^c^^†^	67.34 ± 14.01	<0.0001
Stroop 4 (s)	44.84 ± 5.11 ^a^*^,^^b^^§^	62.07 ± 22.22 ^c^^†^	78.78 ± 23.56	<0.0001
**Long term verbal memory**				
Immediate Recall	12.9 ± 1.7 ^a^^†^^,^^b^^§^	10.4 ± 2.4 ^c^^†^	7.9 ± 3.4	<0.0001
Delayed Recall	13.2 ± 1.5 ^a^^†^^,^^b^^§^	9.8 ± 2.5 ^c^^†^	7.3 ± 3.8	<0.0001
Recognition	14.7 ± 0.5 ^a^^†^^,^^b^^‡^	13.2 ± 1.5	12.8 ± 2.0	0.0008
A1-15	60.3 ± 6.6 ^a^^‡^^,^^b^^§^	48.7 ± 9.8 ^c^^†^	39.6 ± 11.3	<0.0001

GDS: Geriatric Depression Scale, MMSE: Mini-Mental State Examination, DSST: Digit Symbol Substitution Test. Group effect:

^a^ = young vs. older,

^b^ = young vs. CHD patients,

^c^ = older vs. CHD patients,

* = P<0.05,

^†^ = P<0.01,

^‡^ = P<0.001,

^§^ = P<0.0001.

### Cardiopulmonary exercise and hemodynamic parameters

Table 3 describes cardiopulmonary exercise and hemodynamic parameters in all three groups. Both $\dot{V}{\text{O}}_{2}$ peak and cardiac index were significantly higher among young healthy subjects followed by age-matched control and CHD patients. Importantly, $\dot{V}{\text{O}}_{2}$ peak was between 110 and 140% higher relative to age-predicted values depending upon the group, indicating a physically fit sample (all groups). (See the Results section on Cardiopulmonary exercise and hemodynamic parameters of the S1 File for details).

**Table 3:** Cardiopulmonary exercise testing data in young, old healthy controls and CHD patients.

Cardiopulmonary and hemodynamic variables	Young healthy controls (*n* = 22)	Old healthy controls (*n* = 20)	Patients with CHD (*n* = 25)	ANOVA P value
**Rest**				
Resting heart rate (bpm)	61 ± 11	65 ± 8	70 ± 14	0.0536
Resting CI (l/min/m^2^)	3.10 ± 0.52	2.72 ± 0.43	2.77 ± 0.63	0.0778
LVEF (%)	61 ± 7 ^b^^†^	55 ± 7	53 ± 7	0.0071
Rest SBP (mmHg)	116 ± 8 ^b^*	119 ± 14 ^c^^†^	127 ± 16	0.0155
Rest DBP (mmHg)	70 ± 6	72 ± 10	73 ± 8	0.6404
**At peak**				
$\dot{V}{\text{O}}_{2}$ peak (ml/min/LBM)	59 ± 8 ^a^^§^^,^ ^b^^§^	42 ± 6 ^c^*	36 ± 8	<0.0001
% of $\dot{V}{\text{O}}_{2}$ peak predicted (%)	139 ± 20 ^b^^§^	138 ± 17 ^c^^§^	110 ± 22	<0.0001
$\dot{V}{\text{CO}}_{2}$ (ml/min)	4017 ± 961 ^a^^§^^,^^b^^§^	2703 ± 813	2385 ± 837	<0.0001
R.E.R	1.20 ± 0.05	1.15 ± 0.06	1.16 ± 0.09	0.1012
Peak power (Watts)	273 ± 59 ^a^^§^^,^^b^^§^	189 ± 55 ^c^^†^	138 ± 51	<0.0001
$\dot{V}\text{Epeak}$ (l/min)	122 ± 29 ^a^^‡^^,^^b^^§^	88 ± 31	80 ± 24	<0.0001
% of $\dot{V}\text{Epeak}$ predicted (%)	154 ± 26 ^b^^‡^	139 ± 32 ^c^*	119 ± 30	0.0007
$\dot{V}\text{E}$/$\dot{V}{\text{CO}}_{2}$	34 ± 5	32 ± 5	35 ± 5	0.0759
VT (liters)	2.69 ± 0.54 ^b^^‡^	2.42 ± 0.56	2.13 ± 0.41	0.0017
CI max (l/min/m^2^)	10.43 ± 1.90 ^a^^‡^^,^^b^^§^	8.71 ± 1.82 ^c^*	7.44 ± 1.37	<0.0001
Δ CI max (l/min/m^2^)	7.33 ± 1.77 ^a^*^,^^b^^§^	6.01 ± 1.99 ^c^*	4.66 ± 1.23	<0.0001
Peak HR (puls/min)	184 ± 11 ^a^^§^^,^^b^^§^	155 ± 10 ^c^^§^	132 ± 21	<0.0001
Heart rate reserve (%)	97 ± 7 ^a^*^,^^b^^§^	87 ± 11 ^c^^§^	69 ± 18	<0.0001
HRR at 1 min (puls)	- 23 ± 8	-20 ± 6	- 18 ± 7	0.0903
Max SBP (mmHg)	184 ± 18	185 ± 26	177 ± 24	0.4335
Max DBP (mmHg)	74 ± 8	80 ± 11	78 ± 9	0.0903

LVEF: left ventricular ejection fraction, SBP: systolic blood pressure, DBP: diastolic blood pressure, LBM: lean body mass, R.E.R: respiratory exchange ratio, VT: volume tidal, Bf: breathing frequency, CI: cardiac index, ΔCI max = CI max- Resting CI, C(a-v)O_2_: arterio venous difference, SVi: stroke volume index, LCWi: left cardiac work index, SVRi: systemic vascular resistance index, HR: heart rate, HRR: heart rate recovery, Group effect:

^a^ = young vs. older,

^b^ = young vs. CHD patients,

^c^ = older vs. CHD patients,

* = P<0.05,

^†^ = P<0.01,

^‡^ = P<0.001,

^§^ = P<0.0001.

### Left prefrontal NIRS parameters during exercise and recovery

**Fig 2:**
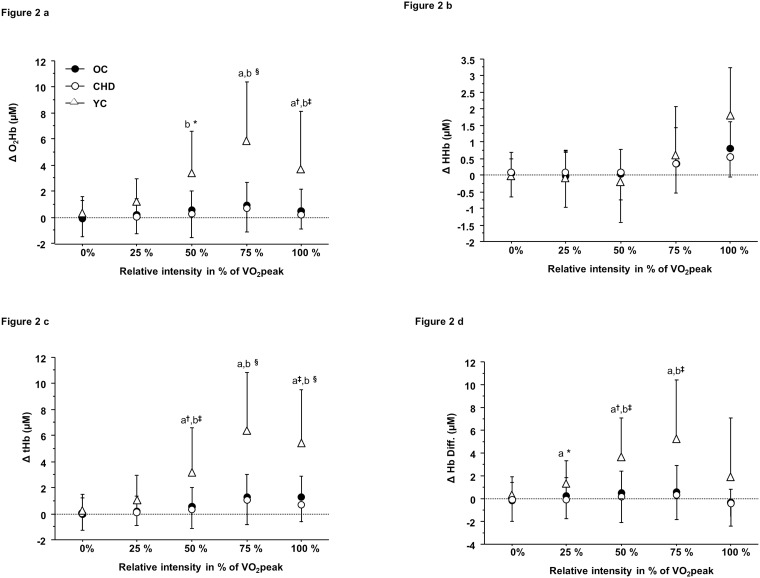
(a-d): Brain NIRS parameters during exercise in young (YC), old healthy controls (OC) and CHD patients (CHD). Post hoc for group effect = a: young vs. old, b = young vs. CHD, * = P<0.05, † = P<0.01, ‡ = P<0.001, § = P<0.0001.

**Fig 3:**
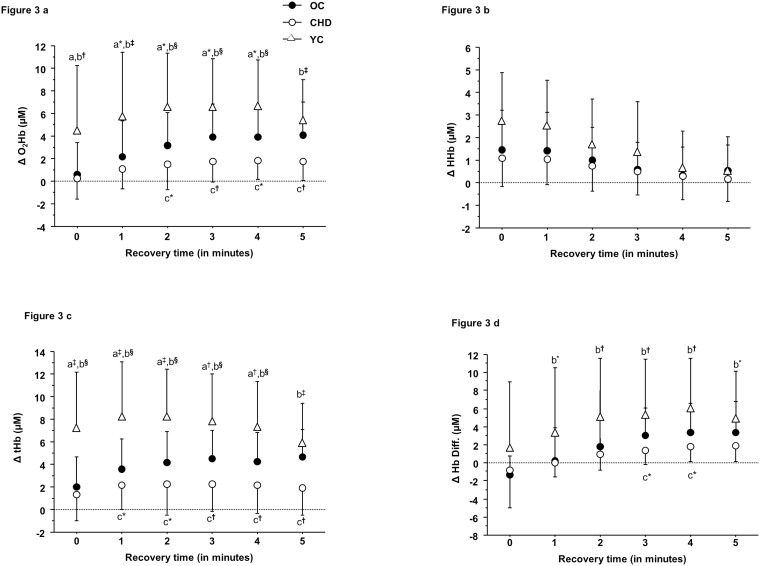
(a-d): Brain NIRS parameters during recovery in young (YC), old healthy controls (OC) and CHD patients (CHD). Post hoc for group effect = a: young vs. old, b = young vs. CHD, c = old vs. CHD, * = P<0.05, † = P<0.01, ‡ = P<0.001, § = P<0.0001.

Figs 2a, 2b, 2c, 2d, 3a, 3b, 3c and 3d describes left prefrontal NIRS parameters during exercise and recovery in all three groups. All parameters were similar at baseline and increased progressively until peak exercise. However, ΔO_2_ Hb and ΔtHb were significantly higher at peak exercise in young healthy controls relative to CHD patients and age-matched healthy controls (P<0.01). (See the Results section on Left prefrontal NIRS parameters during exercise and recovery of the S1 File for details).

### Relationships between $\dot{V}{\text{O}}_{2}$ peak, maximal cardiac index, left prefrontal NIRS and cognitive function parameters

The relationships between $\dot{V}{\text{O}}_{2}$ peak, CI max, left prefrontal NIRS parameters (ΔO_2_ Hb, ΔHHb, ΔtHb, ΔHb diff.) and selected cognitive tasks are described in the Table A and B in S2 File (see the Results section of the S1 File). In summary, statistically significant univariate relationships were observed between all cognitive tasks and both $\dot{V}{\text{O}}_{2}$ peak and CI max, while most cognitive tasks were significantly associated with NIRS parameters.

### Stepwise multiple regression analysis

Table 4(A)–4(D) describes univariate and multivariate analyses used to identify independent predictors of each cognitive composite z score. The relationship between composite z scores representing four cognitive domains (memory, speed of processing, executive functions and verbal memory) and CV risk factors, body composition, exercise and NIRS parameters are presented in Table 4. Briefly, trunk fat mass, education and diabetes were independent predictors of composite working memory score, while maximal total hemoglobin (ΔtHbmax) was the only independent predictor of the speed of processing score. Age and LDL-cholesterol were independent predictors for executive functioning score, whereas $\dot{V}{\text{O}}_{2}$ peak /LBM and gender were independent predictors of the verbal memory score.

**Table 4:** (A-D): Univariate and multivariate analyses used to identify predictors of each cognitive composite z score.

**A: Predictors of working memory composite score**				
Variables	Univariate analysis		Multivariate analysis	
	R	P value	Beta	P value
Age	-0.682	<0.0001		
Education	0.435	0.0006	0.066	0.0314
Height	0.277	0.0321		
Gender	0.145	0.2671		
Obesity	-0.363	0.0044		
Type 2 diabetes	-0.326	0.0108	-0.644	0.0103
Hypertension	-0.419	0.0009		
Dyslipidemias	-0.460	0.0002		
Body mass	-0.164	0.2102		
BMI	-0.413	0.0010		
WC	-0.347	0.0108		
Fat mass	-0.374	0.0045		
Trunk fat mass	-0.491	0.0001	-0.049	0.0014
Fasting glucose	-0.374	0.0067		
Tot chol	0.358	0.0099		
HDL	0.314	0.0245		
LDL	0.279	0.0474		
TG	-0.189	0.1832		
TG/HDL	-0.310	0.0263		
$\dot{V}{\text{O}}_{2}$ peak /LBM	0.587	<0.0001		
$\dot{V}{\text{O}}_{2}$ peak in % of predicted value	0.2167	0.1053		
CImax	0.474	0.0001		
O_2_Hbmax	0.457	0.0003		
O_2_HB recovery	0.345	0.0073		
HHbmax	0.264	0.0429		
HHb recovery	0.164	0.2119		
tHb max	0.528	<0.0001		
tHb recovery	0.451	0.0003		
Hb Diff max	0.404	0.0015		
Hb Diff recovery	0.320	0.0134		
SBP rest	-0.258	0.0484		
DBP rest	-0.059	0.6551		
**B: Predictors of speed of processing composite score**				
Variables	Univariate analysis		Multivariate analysis	
	R	P value	Beta	P value
Age	0.482	0.0001		
Education	-0.325	0.0143		
Height	-0.222	0.0967		
Gender	-0.012	0.9284		
Obesity	0.109	0.4193		
Type 2 diabetes	0.100	0.4558		
Hypertension	0.086	0.5247		
Dyslipidemias	0.292	0.0270		
Body mass	0.093	0.4893		
BMI	0.251	0.0596		
WC	0.171	0.2281		
Fat mass	0.330	0.0156		
Trunk fat mass	0.331	0.0153		
Fasting glucose	0.093	0.5245		
Tot chol	-0.108	0.4566		
HDL	-0.035	0.8082		
LDL	-0.086	0.5545		
TG	-0.012	0.9338		
TG/HDL	0.052	0.7212		
$\dot{V}{\text{O}}_{2}$ peak /LBM	-0.306	0.0204		
$\dot{V}{\text{O}}_{2}$ peak in % of predicted value	0.040	0.7699		
CImax	-0.434	0.0007		
O_2_Hbmax	-0.392	0.0028		
O_2_HB recovery	-0.191	0.1579		
HHbmax	-0.126	0.3529		
HHb recovery	-0.149	0.2720		
tHb max	-0.461	0.0003	-0.038	0.0047
tHb recovery	-0.329	0.0130		
Hb Diff max	-0.354	0.0074		
Hb Diff recovery	-0.065	0.6332		
SBP rest	0.069	0.6128		
DBP rest	0185	0.1703		
**C: Predictors of executive functioning composite score**				
Variables	Univariate analysis	Multivariate analysis		
	R	P value	Beta	P value
Age	0.848	<0.0001	0.035	<0.0001
Education	-0.356	0.0070		
Height	-0.343	0.0088		
Gender	-0.243	0.0683		
Obesity	0.386	0.0030		
Type 2 diabetes	0.172	0.2002		
Hypertension	0.409	0.0016		
Dyslipidemias	0.525	<0.0001		
Body mass	0.052	0.6995		
BMI	0.312	0.0180		
WC	0.305	0.0292		
Fat mass	0.435	0.0011		
Trunk fat mass	0.574	<0.0001		
Fasting glucose	0.309	0.0304		
Tot chol	-0.307	0.0089		
HDL	-0.221	0.1257		
LDL	-0.323	0.0235	-0.284	0.0103
TG	0.274	0.0561		
TG/HDL	0.346	0.0146		
$\dot{V}{\text{O}}_{2}$ peak /LBM	-0.768	<0.0001		
$\dot{V}{\text{O}}_{2}$ peak in % of predicted value	-0.385	0.0033		
CImax	-0.575	<0.0001		
O_2_Hbmax	-0.651	<0.0001		
O_2_HB recovery	-0.268	0.0458		
HHbmax	-0.252	0.0607		
HHb recovery	-0.219	0.1041		
tHb max	-0.707	<0.0001		
tHb recovery	-0.584	<0.0001		
Hb Diff max	-0.570	<0.0001		
Hb Diff recovery	-0.357	0.0069		
SBP rest	0.249	0.0641		
DBP rest	0.119	0.3822		
**D: Predictors of verbal memory composite score**				
Variables	Univariate analysis		Multivariate analysis	
	R	P value	Beta	P value
Age	-0.700	<0.0001		
Education	0.431	0.0006		
Height	0.258	0.0460		
Gender	0.338	0.0082	0.678	0.0419
Obesity	-0.325	0.0112		
Type 2 diabetes	-0.115	0.3798		
Hypertension	-0.238	0.0660		
Dyslipidemias	-0.305	0.0177		
Body mass	-0.378	0.0029		
BMI	-0.346	0.0110		
WC	-0.346	0.0110		
Fat mass	-0.254	0.0581		
Trunk fat mass	-0.400	0.0022		
Fasting glucose	-0.115	0.4203		
Tot chol	0.241	0.0883		
HDL	0.282	0.0447		
LDL	0.134	0.3470		
TG	-0.180	0.2038		
TG/HDL	-0.280	0.0426		
$\dot{V}{\text{O}}_{2}$ peak /LBM	0.656	<0.0001	0.044	0.0002
$\dot{V}{\text{O}}_{2}$ peak in % of predicted value	0.368	0.0047		
CImax	0.337	0.0084		
O_2_Hbmax	0.483	0.0001		
O_2_HB recovery	0.266	0.0413		
HHbmax	0.443	0.0004		
HHb recovery	0.368	0.0041		
tHb max	0.582	<0.0001		
tHb recovery	0.480	0.0001		
Hb Diff max	0.397	0.0018		
Hb Diff recovery	0.335	0.0093		
SBP rest	-0.281	0.0307		
DBP rest	0.024	0.8558		

## Discussion

### Major findings

The main findings of this study are that: 1) CHD patients despite being fit, scored significantly worse on cognitive tests relating to short-term and working memory, processing speed, inhibition and flexibility and long-term verbal memory compared to fit age-matched and young healthy controls, 2) cerebral oxygenation/perfusion during exercise was significantly lower among CHD patients and age-matched controls relative to young controls and 3) executive and memory cognitive functions correlated with $\dot{V}{\text{O}}_{2}$ peak, maximal cardiac index and cerebral oxygenation/perfusion (ΔO_2_ ΔHHb, ΔtHb) during exercise among the entire cohort. To the best of our knowledge, this study is the first to relate executive cognitive functions with simultaneous measures of cardiopulmonary and cardiac function, and cerebral hemodynamics. Our data further support the hypothesis that cardiac function plays a role in the normal decline in cognitive function with aging, and that high fitness alone as a consequence of regular exercise training is insufficient to prevent this decline although it may be slowed.

### Cognitive function in fit cohorts

A large and growing body of evidence highlights the positive relationship that exists between cardiorespiratory fitness and cognitive function in both middle-aged [30] and older healthy adults [31, 32]. Furthermore, higher fitness at a younger age is predictive of better cognitive function later on in life [33, 34]. These data are also consistent with others showing the benefits of exercise training on cognitive function in otherwise healthy individuals [11]. Correlates of this association include structural differences (greater white matter, cerebral blood flow etc.). Few data, however, exist regarding cognitive function in highly fit individuals, and particularly in those with chronic disease. A recent study in elderly marathon runners (mean age 66 years, functional capacity 140% of predicted) demonstrated better cognitive function in one executive function domain (non-verbal fluency assessed via Five Point Test) relative to age-matched controls, while all other executive functions were similar between groups. In a small study in Masters Athletes (n = 12, mean age 72 years), letter and category fluency were significantly better relative to both sedentary elderly and young controls, while other cognitive domains were similar between all three groups [35]. Despite the fact that the prevalence of cognitive decline is higher among patients with CHD or vascular risk factors [36–42], no studies however have evaluated whether fitness level influences cognitive function among such subjects with CHD. Our data would indicate that regular physical activity and high fitness level are insufficient to prevent the “step-down” in cognitive function that occurs from healthy age-matched controls to older patients with CHD. One explanation for this persistence difference could include irreversible vascular changes whereby exercise is no longer able to exert its beneficial effects on the cerebral vasculature. Other potential explanations include a higher prevalence of irreversible brain lesions in patients with CHD such as white matter lesions, brain infarcts and brain atrophy, which also would not be expected to respond to exercise training. Furthermore, CHD patients were already receiving optimal pharmacologic therapy for vascular disease including 92% on statins and 96% on antiplatelet agents and vascular function may have already been at the maximal achievable level.

### Cerebral oxygenation during exercise and recovery

We observed higher cerebral oxygenation (ΔO_2_ Hb) and perfusion (ΔtHb) among healthy subjects during exercise (from 50 to 100% of $\dot{V}{\text{O}}_{2}$ peak) and recovery (0 to 5 min) relative to the two older groups (CHD and age-matched controls). Furthermore, patients with CHD showed evidence of reduced cerebral oxygenation (ΔO_2_ Hb) and perfusion (ΔtHb) during recovery only compared to healthy age-matched controls. Two previous studies demonstrated that aging is associated with a reduction in cerebral perfusion [12, 43] during exercise. The first study (using simultaneous arterial and jugular venous blood gases) showed a similar cerebral oxygen extraction in young and old healthy subjects [43], whereas a second one demonstrated lower right prefrontal cerebral oxygenation and perfusion (ΔO_2_ Hb, ΔtHb) using NIRS in older subjects [12] consistent with our results. The age-related differences in cerebral oxygenation and perfusion we observed could potentially be explained by cerebral vascular aging. This phenomenon includes stiffening of the carotid arteries thereby increasing blood pulsatility in the brain microcirculation, reduced cerebral vascular conductance, brain endothelial dysfunction and reduced brain capillary density [43, 44]. The impact of CVD on cerebral oxygenation and perfusion would appear to depend upon the severity of cardiac dysfunction. For example, one study observed no difference in cerebral exercise oxygenation (ΔO_2_ Hb) or perfusion (ΔtHb) among patients with New York Heart Association (NYHA) class II heart failure and healthy controls, whereas a reduced cerebral oxygenation/perfusion was demonstrated in patients with NYHA class III heart failure vs. the same healthy control group [10]. As well, two other studies demonstrated reduced cerebral oxygenation (ΔO_2_ Hb) and perfusion (ΔtHb) using NIRS during exercise and recovery in patients with valvular heart disease or idiopathic dilated cardiomyopathy with reduced left ventricular ejection fraction (<40%) as compared to older aged-matched healthy controls [8, 9]. Similarly to our results, no differences in cerebral exercise oxygenation (ΔO_2_ Hb) and perfusion (ΔtHb) were observed in heart failure patients (NHYA class II) and healthy controls [10]. We believe that the etiology of heart disease (stable CHD vs. valvular and heart failure), our preserved patient’s aerobic capacity and left ventricular ejection fraction (data not shown) may explain the lack of difference during exercise. In agreement with a previous study in heart failure patients [9], post-exercise cerebral oxygenation (ΔO_2_ Hb) and perfusion (ΔtHb) were reduced in patients with CHD. These results could be attributed to a lower overshoot of cardiac output during recovery in CHD patients vs. older healthy controls [9, 10].

### Cognitive function parameters and their relationships with $\dot{V}{\text{O}}_{2}$ peak, cardiac and cerebral hemodynamics

We found that $\dot{V}{\text{O}}_{2}$ peak, maximal cardiac output (CI max) and cerebral oxygenation and perfusion (ΔO_2_ Hb, ΔtHb) during exercise and, to a lesser degree during recovery, were related to resting cognitive function (short term and working memory, psychomotor speed, cognitive inhibition and flexibility and long term verbal memory) in agreement with previous studies in healthy subjects, heart failure and transplanted patients [10, 11, 45]. The results are also in accordance with the cerebrovascular reserve hypothesis that highlight that better cognitive function relates to better cerebrovascular responses [13]. Moreover, independent predictors for cognitive function (composite score) were also identified. They included education, diabetes, and trunk adiposity for working memory and gender and aerobic fitness for verbal memory. In two studies in obese subjects and heart failure patients, obesity, gender and education were also predictors of memory [46, 47]. In contrast, in a previous work in CHD patients, aerobic fitness ($\dot{V}{\text{O}}_{2}$ peak) was not an independent predictor of memory function [48]. Cerebral perfusion during exercise (tHbmax) independently predicted speed of processing in our study. Previously, resting cerebral perfusion (MRI) was shown not to be related to trail making A (a component of speed of processing score) in multiple linear regression models in older adults with CV disease [14], Finally, we found that age and LDL-cholesterol were independent predictors of executive functioning. Age is a known predictor of executive function in healthy subjects and cardiac patients [11, 46], however we have no clear explanation why LDL-cholesterol remained in the models except as a possible marker of cerebral atherosclerosis. In addition, we are surprised that ($\dot{V}{\text{O}}_{2}$ peak) was not independently related to executive function as shown in other previous studies in healthy subjects and CHD patients [48, 49].

### Limitations

Our study has limitations, including the enrolment of healthy subjects and stable selected CHD patients (mostly men) recruited in a single centre, hence inducing a potential recruitment bias. Patients with CHD were also receiving optimal or near-optimal medical therapy and following a preventive cardiology program. Results may differ in women and in other CHD patients (ex: following acute coronary syndromes) in the real-world setting. As well, cerebral oxygenation and perfusion were assessed non-invasively using NIRS at the left prefrontal area level implicating a very limited spatial resolution and a relatively superficial brain tissue measurement (light penetration ≈ 2.25 cm). Therefore, our results may differ from other more invasive and global measurement of brain oxygenation and perfusion (ex: catheters) or from other brain regions. Another limitation is that cognitive function was only assessed at rest and not during exercise (e.g: Stroop task) in our subjects sample.

### Conclusions

In this study, we demonstrate that fit CHD patients show evidence of significantly reduced resting cognitive functions, particularly with respect to the executive and memory domains, relative to age-matched healthy controls and younger controls. These data indicate that fitness alone does not or cannot prevent the clear step-down in cognitive functions that occur in patients with CHD relative to age-matched healthy controls. Furthermore, while the aging process itself is associated with reduced cognitive function, based upon our results, it is unclear what role reduced cerebral oxygenation/perfusion and cardiopulmonary and hemodynamic responses play in explaining the impairments in cognitive function we observed in our fit CHD cohort. Larger studies using several imaging modalities for evaluating cerebral blood flow and oxygenation/perfusion are required to better understand the potential role of cerebrovascular and neurovascular function in explaining the cognitive impairments we observed in our two aged cohorts. Finally, studies of pharmacological and non-pharmacological treatments known to improve vascular function should be conducted in order to evaluate their potential impact on cognitive functions and assess potential mechanisms. For example, high-intensity interval training was recently shown to have superior effects on $\dot{V}{\text{O}}_{2}$ peak, cardiac output, and cerebral perfusion in patients with chronic heart failure [50] and could be a promising approach to enhance cognitive functions in subjects with CVD.

## Supporting information

S1 File Supplemental text. (DOCX)

S2 File A-B Table. (DOCX)

S3 File Trends Statement checklist.pdf. (PDF)

S4 File Research project and ethical approval. (PDF)

S5 File Deviation protocol COGNEX Plosone.pdf. (PDF)
