# Supplementary material for: Cognitive function in patients with stable coronary heart disease: Related cerebrovascular and cardiovascular responses
Source: PLoS One. 2017 Sep 22;12(9):e0183791. doi: 10.1371/journal.pone.0183791 (PMC5609740; doi:10.1371/journal.pone.0183791)
Supplement: S1 File — (DOCX) [file pone.0183791.s001.docx]

**SUPPLEMENTARY MATERIAL**

**Supplemental Text**

*Inclusion – exclusion criteria*

For healthy controls, inclusion criteria were: age >18 years, no evidence of CHD and no cardiovascular (CV) risk factors. For CHD patients, inclusion criteria was age >18 years and CHD defined as the presence of documented prior myocardial infarction (by history, electrocardiogram, and/or enzyme criteria), prior coronary revascularization, angiographic evidence of atherosclerosis or documented myocardial ischemia on myocardial scintigraphy [15] . The CV risk factors were defined as followed [15]. Diabetes was defined as a prior diagnosis of diabetes along with a fasting glucose >7.1 mmol/L and an HbA_1c_ level >0.06 or a treatment with an hypoglycemic agent. Hypertension was defined as a prior diagnosis of hypertension with blood pressure >130/85 mm Hg or antihypertensive treatment. Active smoking was defined as smoking ≥1 cigarette, cigar, or pipe per day. Obesity was defined by a BMI over 30 kg/m^2^. Dyslipidemia was defined as total cholesterol ≥6.2 mmol/L, low-density lipoprotein cholesterol ≥ 4.2 mmol/L, or a total/high-density lipoprotein cholesterol ratio ≥4.7 or statin treatment. Exclusion criteria were: recent acute coronary syndrome (<3 months), significant resting ECG abnormality, history of ventricular arrhythmias or congestive heart failure, uncontrolled hypertension, recent bypass surgery intervention <3 months, recent percutaneous transluminal coronary angioplasty <6 months, left ventricular ejection fraction <45%, pacemaker or implantable cardioverter defibrillator, recent modification of medication <2 weeks, respiratory/cerebral diseases and musculoskeletal conditions making exercise on ergocycle not indicated [15]. For cognitive testing, exclusion criteria were: a mini mental status exam (MMSE) score <26, and/or a geriatric depression scale >10, as well as an history of neurological (i.e. stroke, dementia, Alzheimer’s disease) or psychiatric (i.e. bipolar disorder, schizophrenia) problems [16,17].

**Results**

**Clinical characteristics**

The prevalence of cardiovascular risk factors was higher in patients with CHD compared to healthy controls, particularly for dyslipidemia (80%), hypertension (40%), obesity (44%) and diabetes (28%). As expected, medication prevalence was also higher in patients with CHD (vs. older controls) with a large proportion of patients taking antiplatelets agents (96%), statins (92%), calcium channels blockers and ACE inhibitors (36 and 32 %). As well, compared to healthy controls, patients with CHD had a higher body mass, BMI, WC and fat mass (P<0.05). Young healthy controls had a lower fat mass compared to the 2 other groups (P<0.05). Regarding blood analysis, patients with CHD had higher levels of fasting glycemia, triglycerides, TG/HDL ratio and lower levels of total, LDL and HDL cholesterol (P<0.05) vs. the 2 healthy groups.

**Cognitive function parameters**

Young healthy subjects had higher Forward Span score vs. the 2 other groups (old and CHD) and higher Backward Span score vs. patients with CHD (P<0.001). Patients with CHD had a lower short term and working memory scores (Forward and Backward Span) vs. older healthy subjects (P<0.01). Young healthy subjects had lower perceptual abilities and processing speed scores (Trail A, Stroop 1 and 2, P<0.01) and higher DSST score (P<0.0001) vs. the 2 other groups (old and CHD). Patients with CHD had a lower DSST score vs. older healthy subjects (P<0.01). Young healthy subjects had lower cognitive inhibition and flexibility scores (Trail B, Stroop 3 and 4) vs. the 2 other groups (old and CHD) (P<0.05). Patients with CHD had a higher cognitive inhibition and flexibility scores (Trail B, Stroop 3 and 4) vs. older healthy subjects (P<0.01). Young healthy subjects had higher long term verbal memory scores (immediate and delayed recall, recognition and A1-15) vs. the 2 other groups (old and CHD) (P<0.01). Patients with CHD had lower long term verbal memory scores (immediate and delayed recall, A1-15) vs. older healthy subjects (P<0.01).

**Cardiopulmonary exercise testing parameters**

Patients with CHD had a higher SBP (P<0.05) vs. the 2 healthy groups. Young healthy subjects had a higher**uptake and power at ventilatory threshold (VT) and peak exercise vs. the 2 other groups (old and CHD) (P<0.0001). Patients with CHD had a lower **peak, % of **predicted, peak power, **and power VT vs. older healthy controls (P<0.05). Young healthy subjects had a higher **, **peak and Bf vs. the 2 other groups (old and CHD) (P<0.01). Patients with CHD had a lower % of **peak predicted vs. older healthy controls (P<0.05). At peak exercise, young healthy subjects had a higher CI max, LCWi, SVRi, HR and heart rate reserve vs. the 2 other groups (old and CHD) (P<0.05). Patients with CHD had a lower CI max, peak HR and hear rate reserve vs. older healthy controls (P<0.05).

**Left prefrontal NIRS parameters during exercise and recovery**

During exercise, young healthy subjects had higher values for O_2_ Hb and tHb (µM) (from 50% to 100% of **peak, P<0.05) and higher values for Hb diff. (µM) (from 25% to 100% of **peak, P<0.05) vs. the 2 other groups (old and CHD). During recovery, young healthy subjects had higher values for O_2_ Hb and tHb (µM) (from 0 to 5 min, P<0.05) vs. the 2 others groups (old and CHD). Young healthy subjects had higher values for Hb diff. (µM) (from 1 to 5 min, P<0.05) vs. patients with CHD. During recovery, patients with CHD had a lower values for O_2_ Hb (from 2 to 5 min, P<0.05), tHb (µM) (from 1 to 5 min, P<0.05) and Hb diff. (at 3 and 4 min, P<0.05) vs. old healthy controls.

**Relationships between **peak, maximal cardiac index, left prefrontal NIRS and cognitive function parameters**

During exercise, **peak was significantly related to left prefrontal NIRS parameters (R=0.41 to 0.66, P<0.001) as well as CI max (R=0.36 to 0.56, P<0.01). Similarly, **peak was significantly related to all selected cognitive items (R= -0.67 to 0.67, P<0.01) as well as CI max (R= -0.42 to 0.50, P<0.01). Finally, ΔO_2_ Hb and ΔtHb were related to all selected cognitive function items (R= -0.49 to 0.55, P<0.05). ΔHHb was related to quasi all selected cognitive function items (R= -0.42 to 0.53, P<0.05), excepted with Backward span (P=0.06). ΔHb diff. was related to quasi all selected cognitive function items (R= -0.44 to 0.47, P<0.05), excepted with Backward span (P=0.06) and Immediate recall (P=0.07).

**Table S1 (A and B):** Relationship between VO_2_peak, cardiac index, brain maximal NIRS variables **(Δ)** and cognitive function in all subjects.

| **A) All patients (n=67)** | | | | |
| --- | --- | --- | --- | --- |
| **Exercise** | **$\mathop{V}\limits^{.}$O_2_ peak (ml/min/LBM)** | | **CI max (l/min/m^2^)** | |
| Forward Span | R=0.54, P<0.0001 | | R=0.40, P=0.0024 | |
| Backward Span | R=0.34, P=0.0091 | | R=0.40, P=0.0025 | |
| DSST | R=0.67, P<0.0001 | | R=0.46, P=0.0003 | |
| Trail B (s) | R= -0.61, P<0.0001 | | R= -0.42, P=0.0013 | |
| Stroop 3 (s) | R= -0.67, P<0.0001 | | R= -0.39, P=0.0026 | |
| Stroop 4 (s) | R= -0.58, P<0.0001 | | R= -0.39, P=0.0021 | |
| Immediate Recall | R= 0.57, P<0.0001 | | R=0.41, P=0.0015 | |
| Delayed Recall | R= 0.64, P<0.0001 | | R=0.50, P<0.0001 | |
| A1-15 | R= 0.62, P<0.0001 | | R=0.45, P=0.0005 | |
| **B) All patients (n=67)** | | | | |
| **Exercise** | **Δ O_2_ Hb (µM)** | **Δ HHb (µM)** | **Δ tHb (µM)** | **Δ Hb diff. (µM)** |
| Forward Span | R=0.37, P=0.0041 | R=0.27, P=0.0388 | R=0.43, P=0.0008 | R=0.34, P=0.0091 |
| Backward Span | R=0.32, P=0.0145 | R=0.24, P=0.0686 | R=0.33, P=0.0113 | R=0.30, P=0.0214 |
| DSST | R=0.55, P<0.0001 | R=0.40, P=0.0022 | R=0.65, P<0.0001 | R=0.47, P=0.0002 |
| Trail B (s) | R= -0.49, P<0.0001 | R= -0.32, P=0.0148 | R= -0.57, P<0.0001 | R= -0.44, P=0.0006 |
| Stroop 3 (s) | R= -0.49, P<0.0001 | R= -0.42, P=0.0010 | R= -0.59, P<0.0001 | R= -0.42, P=0.0011 |
| Stroop 4 (s) | R= -0.47, P=0.0002 | R= -0.24, P=0.0637 | R= -0.51, P<0.0001 | R= -0.42, P=0.0011 |
| Immediate Recall | R=0.32, P=0.0158 | R=0.52, P<0.0001 | R=0.43, P=0.0007 | R=0.23, P=0.0788 |
| Delayed Recall | R=0.42, P=0.0010 | R=0.53, P<0.0001 | R=0.53, P<0.0001 | R=0.34, P=0.0102 |
| A1-15 | R=0.49, P=0.0005 | R=0.50, P<0.0001 | R=0.54, P<0.0001 | R=0.36, P=0.0052 |
| **Recovery** |  |  |  |  |
| Forward Span | R=0.24, P=0.0667 | R=0.28, P=0.0373 | R=0.39, P=0.0023 | R=0.37, P=0.0046 |
| Backward Span | R=0.12, P=0.3715 | R=0.17, P=0.2080 | R=0.39, P=0.0029 | R=0.26, P=0.0529 |
| DSST | R=0.25, P=0.0644 | R=0.38, P=0.0033 | R=0.60, P<0.0001 | R=0.38, P=0.0039 |
| Trail B (s) | R= -0.17, P=0.2107 | R= -0.31, P=0.0177 | R= -0.54, P<0.0001 | R= -0.31, P=0.0200 |
| Stroop 3 (s) | R= -0.30, P=0.0226 | R= -0.39, P=0.0024 | R= -0.55, P<0.0001 | R= -0.31, P=0.0191 |
| Stroop 4 (s) | R= -0.22, P=0.1041 | R= -0.24, P=0.0667 | R= -0.44, P=0.0005 | R= -0.26, P=0.0491 |
| Immediate Recall | R=0.33, P=0.0122 | R=0.47, P=0.0002 | R=0.38, P=0.0039 | R=0.20, P=0.1330 |
| Delayed Recall | R=0.27, P=0.0405 | R=0.48, P=0.0001 | R=0.48, P=0.0001 | R=0.26, P=0.0484 |
| A1-15 | R=0.30, P=0.0257 | R=0.48, P=0.0002 | R= 0.51, P<0.0001 | R=0.31, P=0.0175 |

Δ = delta, Δ values were calculated by subtracting baseline with maximal value.
