# Supplementary material for: Cognitive function in patients with stable coronary heart disease: Related cerebrovascular and cardiovascular responses
Source: PLoS One. 2017 Sep 22;12(9):e0183791. doi: 10.1371/journal.pone.0183791 (PMC5609740; doi:10.1371/journal.pone.0183791)
Supplement: S2 File — (DOCX) [file pone.0183791.s002.docx]

**Table A and B:** Relationship between VO_2_peak, cardiac index, brain maximal NIRS variables **(Δ)** and cognitive function in all subjects.

| **A) All patients (n=67)** | | | | |
| --- | --- | --- | --- | --- |
| **Exercise** | **$\mathop{V}\limits^{.}$O_2_ peak (ml/min/LBM)** | | **CI max (l/min/m^2^)** | |
| Forward Span | R=0.54, P<0.0001 | | R=0.40, P=0.0024 | |
| Backward Span | R=0.34, P=0.0091 | | R=0.40, P=0.0025 | |
| DSST | R=0.67, P<0.0001 | | R=0.46, P=0.0003 | |
| Trail B (s) | R= -0.61, P<0.0001 | | R= -0.42, P=0.0013 | |
| Stroop 3 (s) | R= -0.67, P<0.0001 | | R= -0.39, P=0.0026 | |
| Stroop 4 (s) | R= -0.58, P<0.0001 | | R= -0.39, P=0.0021 | |
| Immediate Recall | R= 0.57, P<0.0001 | | R=0.41, P=0.0015 | |
| Delayed Recall | R= 0.64, P<0.0001 | | R=0.50, P<0.0001 | |
| A1-15 | R= 0.62, P<0.0001 | | R=0.45, P=0.0005 | |
| **B) All patients (n=67)** | | | | |
| **Exercise** | **Δ O_2_ Hb (µM)** | **Δ HHb (µM)** | **Δ tHb (µM)** | **Δ Hb diff. (µM)** |
| Forward Span | R=0.37, P=0.0041 | R=0.27, P=0.0388 | R=0.43, P=0.0008 | R=0.34, P=0.0091 |
| Backward Span | R=0.32, P=0.0145 | R=0.24, P=0.0686 | R=0.33, P=0.0113 | R=0.30, P=0.0214 |
| DSST | R=0.55, P<0.0001 | R=0.40, P=0.0022 | R=0.65, P<0.0001 | R=0.47, P=0.0002 |
| Trail B (s) | R= -0.49, P<0.0001 | R= -0.32, P=0.0148 | R= -0.57, P<0.0001 | R= -0.44, P=0.0006 |
| Stroop 3 (s) | R= -0.49, P<0.0001 | R= -0.42, P=0.0010 | R= -0.59, P<0.0001 | R= -0.42, P=0.0011 |
| Stroop 4 (s) | R= -0.47, P=0.0002 | R= -0.24, P=0.0637 | R= -0.51, P<0.0001 | R= -0.42, P=0.0011 |
| Immediate Recall | R=0.32, P=0.0158 | R=0.52, P<0.0001 | R=0.43, P=0.0007 | R=0.23, P=0.0788 |
| Delayed Recall | R=0.42, P=0.0010 | R=0.53, P<0.0001 | R=0.53, P<0.0001 | R=0.34, P=0.0102 |
| A1-15 | R=0.49, P=0.0005 | R=0.50, P<0.0001 | R=0.54, P<0.0001 | R=0.36, P=0.0052 |
| **Recovery** |  |  |  |  |
| Forward Span | R=0.24, P=0.0667 | R=0.28, P=0.0373 | R=0.39, P=0.0023 | R=0.37, P=0.0046 |
| Backward Span | R=0.12, P=0.3715 | R=0.17, P=0.2080 | R=0.39, P=0.0029 | R=0.26, P=0.0529 |
| DSST | R=0.25, P=0.0644 | R=0.38, P=0.0033 | R=0.60, P<0.0001 | R=0.38, P=0.0039 |
| Trail B (s) | R= -0.17, P=0.2107 | R= -0.31, P=0.0177 | R= -0.54, P<0.0001 | R= -0.31, P=0.0200 |
| Stroop 3 (s) | R= -0.30, P=0.0226 | R= -0.39, P=0.0024 | R= -0.55, P<0.0001 | R= -0.31, P=0.0191 |
| Stroop 4 (s) | R= -0.22, P=0.1041 | R= -0.24, P=0.0667 | R= -0.44, P=0.0005 | R= -0.26, P=0.0491 |
| Immediate Recall | R=0.33, P=0.0122 | R=0.47, P=0.0002 | R=0.38, P=0.0039 | R=0.20, P=0.1330 |
| Delayed Recall | R=0.27, P=0.0405 | R=0.48, P=0.0001 | R=0.48, P=0.0001 | R=0.26, P=0.0484 |
| A1-15 | R=0.30, P=0.0257 | R=0.48, P=0.0002 | R= 0.51, P<0.0001 | R=0.31, P=0.0175 |

Δ = delta, Δ values were calculated by subtracting baseline with maximal value.
