# Supplementary material for: Cognitive function in patients with stable coronary heart disease: Related cerebrovascular and cardiovascular responses
Source: PLoS One. 2017 Sep 22;12(9):e0183791. doi: 10.1371/journal.pone.0183791 (PMC5609740; doi:10.1371/journal.pone.0183791)
Supplement: S4 File — (PDF) [file pone.0183791.s004.pdf]

FORMULAIRE DE SOUMISSION D'UN PROJET DE RECHERCHE

VERSION no. 2 datée du 23-04-2012

PROJET *COGNEX*

**CHERCHEUR PRINCIPAL :**	**Dr Anil Nigam (MD, Ms. C)**
**COLLABORATEURS :**	Dr Mathieu Gayda (Ph.D)<br>Dr Vincent Gremeaux (MD, Ms. C)<br>Dr Martin Juneau (M.D)<br>Dr Frédéric Lesage (Ph.D)<br>Dr Eric Thorin (Ph.D)<br>Pr Louis Bherer (Ph.D)<br>Mr Saïd Mekary (Ms.C)<br>Dr Mélanie Renaud (Ph.D)<br>Dr Jean Lambert (Ph.D)
**TITRE DU PROJET :**	Oxygénation cérébrale, débit cardiaque, performance cognitive et exercice chez les patients présentant un syndrome métabolique, une maladie coronarienne, ou une insuffisance cardiaque.<br>Cerebral Oxygenation, Cardiac Output,COGnitive function, and EXercise in patients with metabolic syndrome, coronary heart disease and chronic heart failure.

☒ Nouveau projet
☐ Sous-étude - Si oui, indiquer le numéro de l'étude principale :

☒ Institutionnel
☐ Multicentrique - Si oui, indiquer le nombre de centres au Québec :

Projet multicentrique du Ministère :
☐ Oui (Compléter ce document **en entier** ainsi que le formulaire M-EVAL)
☒ Non (Compléter ce document en entier)

TYPE DE PROJET

☒ Essai clinique	☐ Banque de données / matériel bio / les deux (B)
☐ Pharmacologique	☐ Registre
☐ Épidémiologique (ÉPI)	☐ Génétique / Pharmacogénomique (G)
☐ Psychosociale (PSY)	☐ Sciences humaines
☐ Nouvelles technologies (NT)	☐ Cellules souches
☐ Dossier uniquement (D)	☐ Autres (A) :

PROJETS QUI RELÈVENT DE SANTÉ CANADA

Uniquement si applicable, indiquez la ou les catégories auxquelles le projet appartient (cochez la ou les cases correspondantes) :

☐ Recherche qui relève de la compétence de Santé Canada (ex : essai clinique)

☐ Médicament expérimental.

Précisez : ☐ Phase I ☐ Phase II ☐ Phase III ☐ Phase IV

(S'il s'agit d'une phase I, soumettre les informations précises sur les recherche faites sur les animaux)

☐ Phase IIB ☐ Phase IIIB ☐ Autre, spécifiez :

☐ Instrument médical. Précisez : ☐ Classe I ☐ Classe II ☐ Classe III ☐ Classe IV

☐ Produit de santé naturel

☐ Produit radiopharmaceutique **(Si oui, remplir le formulaire de soumission d'un projet de recherche avec usage de produit radioactif chez l'humain)**

☐ Randomisé ☐ Ouvert ☐ En double insu ☐ En simple insu

☐ Placebo ☐ Agent actif comparatif

Quelle est la situation qui s'applique en l'espèce :

☐ Le promoteur ou le fabricant a obtenu l'autorisation de Santé Canada (ex : lettre de non-objection (NOL), avis d'autorisation). **Si oui soumettre ce document.**

☐ Le promoteur ou le fabricant attend la décision de Santé Canada.

☐ Le promoteur ou le fabricant n'a pas déposé de demande à Santé Canada.

☐ L'autorisation de Santé Canada n'est pas nécessaire **(Joignez la lettre de Santé Canada en attestant lorsqu'applicable)**

Le médicament, le produit biochimique, le produit de santé naturel ou le dispositif médical expérimental est-il assujetti à la réglementation de la FDA ?

Date du début du projet :	Octobre 2010
Date de la fin du projet :	Octobre 2013
Durée prévue :	3 ans
Durée prévue de la participation des sujets de recherche pressentis :	4 à 20 semaines

FINANCEMENT

☐ IRSC ☐ FRSQ

☐ FMCQ ☐ NIH

☒ Autre, spécifiez : Fonds des Dr Juneau, Nigam, et Bherer

Centre de coordination scientifique :	Centre EPIC
Nom du coordonnateur de l'étude :	Dr Mathieu Gayda
Nombre de centres participants :	1
Nom du chercheur principal à l'ICM :	Dr Mathieu Gayda
Nombre total de patients dans l'étude :	80
Nombre de patients à l'ICM :	
Nombre de patients provenant de la biobanque de l'ICM :<br>(Compléter la section prévue à cet effet, à la fin du formulaire *Annexe 1 : Utilisation de la biobanque aux fins du projet*).	0
Département / Service où s'effectuera le projet :	Centre EPIC
Laboratoire où s'effectueront les analyses reliées au projet :	Centre EPIC

JUSTIFICATION SCIENTIFIQUE DU PROJET

La diminution des performances cognitives est un problème de santé publique dans la population Canadienne vieillissante, et expose au risque d'altérations cognitives ultérieures. Les principaux facteurs de risques cardiovasculaires, dont la sédentarité, ont également été identifiés comme facteurs de risque de diminution de la performance cognitive. Les mécanismes physiopathologiques sous jacents sont encore discutés, mais parmi les hypothèses avancées, on trouve la baisse du débit cardiaque et l'hypoperfusion cérébrale.

Plusieurs études observationnelles ont montré que l'activité physique régulière induit un effet protecteur sur le déclin de la fonction cognitive chez les sujets sains. Par ailleurs, chez les sujets coronariens, les capacités d'effort sont associées à la performance cognitive. Là encore, les mécanismes par lesquels l'entraînement physique améliore le fonctionnement cérébral restent débattus. Un des mécanismes potentiel serait l'amélioration du fonctionnement vasculaire cérébral et de la perfusion induits par l'exercice. Des travaux ont utilisé la technique de spectroscopie proche de l'infra-rouge (NIRS pour Near InfraRed Spectroscopy) pour étudier l'oxygénation cérébrale pendant une épreuve d'effort maximale, chez des sujets sains et pathologiques. Chez des patients coronariens et insuffisants cardiaques, il a été montré que le profil d'oxygénation cérébrale à l'effort était différent de celui de sujets sains, avec une stabilisation ou une baisse d'oxygénation dès le début de l'effort. De plus, une baisse importante de l'oxygénation cérébrale à l'effort représente un facteur de risque indépendant de mortalité d'origine cardiaque chez les patients coronariens. Cependant, les relations entre l'oxygénation cérébrale à l'effort et la performance cognitive n'ont jamais été étudiées.

Nous proposons donc d'étudier la relation entre performance cognitive, débit cardiaque et oxygénation cérébrale à l'effort, chez des patients présentant un syndrome métabolique, une pathologie coronarienne, ou une insuffisance cardiaque chronique stabilisée :

1/ au cours d'un exercice aigu, dans le but de mieux comprendre les mécanismes de l'impact positif de l'exercice physique sur les performances cognitives;

2/ pendant et après une période d'entraînement de 12 semaines, selon 2 modalités : Entraînement Continu d'Intensité Modérée (ECIM) ; ou Entraînement par Intervalles à Haute Intensité (EIHI). Le but de ce deuxième volet est de déterminer le type d'entraînement optimal pour l'amélioration des performances cognitives chez ces sujets, pour optimiser l'amélioration de leur qualité de vie.

DESCRIPTION SOMMAIRE DU PROJET

Objectif : L'objectif de ce travail est

1/ d'étudier la relation entre la performance cognitive, l'oxygénation cérébrale, et le débit cardiaque à l'effort, chez des patients présentant un syndrome métabolique, une pathologie coronarienne, ou une insuffisance cardiaque chronique stabilisée pendant une épreuve d'effort maximale (volet 1)
2/ d'étudier l'évolution de ces paramètres après une période d'entraînement de 12 semaines, selon 2 modalités : Entraînement Continu d'Intensité Modérée (ECIM) ; ou Entraînement par Intervalles à Haute Intensité (EIHI) (volet 2).

Plan expérimental:

Conception: il s'agit d'une étude monocentrique comportant un premier volet descriptif, puis un second volet interventionnel randomisé en simple insu.

Population:
Volet 1: 20 sujets présentant une pathologie coronarienne stable, 20 sujets présentant un syndrome métabolique, et 20 présentant une insuffisance cardiaque chronique stable. Un groupe témoin de 20 sujets sains appariés (âge et sexe), indemnes de toute pathologie cardiaque et ne présentant pas de syndrome métabolique, sera également recruté. Les patients présentant des troubles anxieux ou dépressifs sévères pouvant interférer avec les performances cognitives seront exclus.
Volet 2: 20 sujets présentant une pathologie coronarienne stable, 20 sujets présentant un syndrome métabolique, et 20 sujets présentant une insuffisance cardiaque chronique stable.

Interventions et mesures:

Volet 1: les patients auront 4 rendez-vous au centre EPIC. Au cours de la première visite, le patient aura un bilan biologique à jeun (cholestérol total, HDL et LDL cholestérol, triglycérides, insuline, glycémie, HbA1c, CRP, Angiopoïétine-like 2), une mesure de la composition corporelle par impédance, et une mesure de la réactivité vasculaire (cérébrale et musculaire) utilisant la technique de spectroscopie dans le proche infrarouge (NIRS). Le bilan cognitif sera réalisé lors de la 2ème visite. La Troisième visite consistera en une épreuve d'effort maximale sur vélo avec enregistrement ECG, du débit cardiaque de manière non invasive par bio-impédance, de l'oxygénation cérébrale par la NIRS, et analyse des gaz expiratoires. Au cours de la quatrième visite sera effectué un court test d'effort sous-maximal rectangulaire comportant 2 périodes, à des puissances inférieures et supérieures au seuil de chute de l'oxygénation cérébrale. Un bref test cognitif sera réalisé au cours de ces 2 périodes.

Volet 2: les patients bénéficieront d'une période d'entraînement de 12 semaines, à raison de 3 à 5 séances par semaine, selon 2 modalités choisies aléatoirement: Entraînement Continu d'Intensité Modérée (ECIM) ; ou Entraînement par Intervalles à Haute Intensité (EIHI). Au terme de cette période, ils auront 4 nouveaux rendez-vous comportant les mêmes tests que lors du volet 1.

Critère primaire:
Volet 1: relation entre performance cognitive et variation de l'oxygénation cérébrale à l'effort.
Volet 2: Amélioration des performances cognitive après l'entraînement.

Critères secondaires:
Volet 1:
Corrélations entre performance cognitive et VO2 pic, débit cardiaque, oxygénation cérébrale, et réactivité cérébro-vasculaire.
Corrélations entre la réactivité vasculaire musculaire et cérébrale.
Volet 2
Corrélations entre les améliorations des performance cognitives et de la VO2 pic, du débit cardiaque, de l'oxygénation cérébrale, et de la réactivité cérébro-vasculaire.
Comparaison de l'amélioration des performances cognitives, de la VO2 pic, du débit cardiaque, de l'oxygénation cérébrale, et de la réactivité cérébro-vasculaire entre les 2 modalités d'entraînement.

Hypothèses:

Volet 1: nous prévoyons que la performance cognitive sera plus basse chez les sujets présentant une plus faible augmentation - ou une diminution - de l'oxygénation cérébrale à l'effort, et que ces performances seront également liées à la VO2pic et au débit cardiaque. Nous formulons également l'hypothèse que la performance cognitive baissera au cours de l'effort, et que cette baisse apparaîtra pour une intensité proche du seuil de chute de l'oxygénation cérébrale.
Volet 2: Nous formulons les hypothèses suivantes: (1) l'entraînement améliore les performances cognitives dans ces 3 populations; et (2) l'entraînement par intervalles (EIHI) permet une amélioration supérieure des performances cognitives, de la VO2 pic, du débit cardiaque, et de la réactivité cérébro-vasculaire.

ÉTATS FINANCIERS

Total des fonds requis pour le projet : **0,00 $**

DÉPENSES ESTIMÉES POUR 1 AN :

1. Personnel :

Nom: Julie Lalongé
Fonction: Technicienne ECG
Heures / semaine : 7
Coût annuel : 11760,00 $

Nom: Heidi Claveau
Fonction: Infirmière
Heures / semaine : 7
Coût annuel : 11760,00 $

Nom
Fonction: Kinésiologue
Heures / semaine : 4
Coût annuel : 9 600,00 $

Nom
Fonction
Heures / semaine :
Coût annuel : 0,00 $

Total coûts personnels :33 120,00 $/an

2. Appareillages et équipements nouveaux nécessaires (svp, inclure copie des estimations) :

Aucun

Coût : 0,00 $

3. Fournitures et services (réactif, analyses de laboratoire, papeterie, animaux, etc.)

- Analyse laboratoire de biochimie de l'ICM

  - Visite 1 Volet 1:

Cholestérol total, HDL cholestérol, LDL cholestérol, Triglycérides, Glycémie à jeun, Insulinémie, HbA1c;
C-terminal BNP pour les patients insuffisants cardiaques

soit 13.9 $ pour les sujets sains, syndrome métabolique, et coronariens × 60 sujets, soit un total de 834 $
soit 47.9 $ pour les sujets insuffisants cardiaques, × 20 sujets, soit un total de 958 $

  - Volet 2

  - Visite 1 volet 2

Cholestérol total, HDL cholestérol, LDL cholestérol, Triglycérides, Glycémie à jeun, Insulinémie, HbA1c;
C-terminal BNP pour les patients insuffisants cardiaques

soit 13.9 $ pour les sujets sains, syndrome métabolique, et coronariens × 60 sujets, soit un total de 834 $
soit 47.9 $ pour les sujets insuffisants cardiaques, × 20 sujets, soit un total de 958 $

Angiopoïétine-like 2 à la visite 1 du Volet 1 et du volet 2, soit 300$ par patient du volet 2, × 60 soit un total de 18000$

- Consommables:
électrodes ECG, électrode bio-impédance, papier ECG, Tegaderm, tubes de sang: 5000$

**Coût :**	**26 584,00 $**

4. Nouveaux espaces requis :
   Aucun

**Coût :**	**0,00 $**

5. Autres dépenses :
   Tests cardio-pulmonaires: 100$ par patient, ×80, soit un total de 8000$

   Compensation: 10$/patients, soit un total de 800$

**Coût :**	**8 800,00 $**
**Total des fonds requis pour l'année :**	**68 504,00 $**

Des fonds pour ce projet ont-ils été demandés à d'autres sources ? Oui

Si oui, spécifier les organismes : IRSC

**Les montants requis :**	**100 000,00 $**

Des fonds ont-ils été obtenus d'autres sources ? Non

Si oui, spécifier les organismes :

**Les montants requis :**	**0,00 $/pt**

FACTURATION DES FRAIS ENTOURANT LA RECHERCHE CLINIQUE

☐ Recherche initiée par l'investigateur

☐ Recherche subventionnées par un organisme reconnu par le FRSQ

Veuillez indiquer le nom de l'organisme et de la personne à qui acheminer les factures suivantes, si applicable.

1. **Frais d'éthique :**

   Compagnie :

   Nom de la personne :

   Titre d'emploi :

   Adresse :

   Téléphone :

   Courriel :

2. **Frais de pharmacie (si différent) :**

   Compagnie :

   Nom de la personne :

   Titre d'emploi :

   Adresse :

   Téléphone :

   Courriel :

3. **Frais d'archivage (si différent) :**

   Compagnie :

   Nom de la personne :

   Titre d'emploi :

   Adresse :

   Téléphone :

   Courriel :

SIGNATURES du chercheur principal, des collaborateurs et du chef de département

Date : ______________________

Signature des collaborateurs :

1. **Dr Mathieu Gayda, Ph.D**
2. **Dr Vincent Grémeaux, MD, Ms.C**
3. **Dr Martin Juneau, MD**
4. **Dr Frédérique Lesage PhD**
5. **Dr Éric Thorin PhD**
6. **Dr Louid Bherer PhD**

Dr Normand Racine
Signature du chef de département

ENGAGEMENT DU CHERCHEUR PRINCIPAL À DIVULGUER TOUT CONFLIT D'INTÉRÊT SELON LA POLITIQUE ÉTABLIE

Je soussigné, m'engage à divulguer tout conflit d'intérêt selon la politique établie à l'Institut de Cardiologie de Montréal. (Référence Manuel des politiques et méthodes de l'ICM – DG-02 – Voir annexe 1 si nécessaire)

Dr Anil Nigam Date
Signature du chercheur principal

DÉCLARATION DE TRANSMISSION DES INFORMATIONS PERSONNELLES DU CHERCHEUR PRINCIPAL

J'autorise l'établissement à transmettre aux autorités compétentes des renseignements nominatifs me concernant en présence d'un cas avéré de manquement à l'intégrité ou à l'éthique ou d'une plainte fondée.

Dr Anil Nigam Date
Signature du chercheur principal

OBJECTIFS DU PROJET

1. Objectif principal :

Etudier dans une population de sujets sains, ou présentant un syndrome métabolique, une pathologie coronarienne, ou une insuffisance cardiaque:

Volet 1: la relation entre la performance cognitive et l'oxygénation cérébrale à l'effort

Volet 2: les effets de l'entraînement sur la performance cognitive et l'oxygénation cérébrale à l'effort.

2. Objectif(s) secondaire(s) :

Volet 1:
1/ Recherche de corrélations entre performance cognitive et VO2 pic, débit cardiaque, oxygénation cérébrale, et réactivité cérébro-vasculaire.
2/ Etudier la reproductibilité du Breath Holding Index (BHI= mesure de la réactivité cérébro-vasculaire) dans cette population.
3/ Etudier les effets de l'intensité de l'effort sur la performance cognitive.

Volet 2:
1/ Rechercher de corrélations entre les améliorations de capacités cognitives et de VO2 pic, débit cardiaque, et réactivité cérébro-vasculaire.
2/ Comparer l'amélioration des performances cognitives et de la VO2 pic, du débit cardiaque, de l'oxygénation cérébrale, et de la réactivité cérébro-vasculaire entre les 2 groupes.

RELEVÉ PERTINENT DE LA LITTÉRATURE SUR LE SUJET

Epidemiology cognitive impairment and dementia in Canada.

Elderly Canadian adults over 65 years old were representing 13.1 % of the total Canadian population in 2005 (Turcotte M et al. 2006, Statistics Canada). With the worldwide ageing of the population in developed countries, elderly adults over 65 years will represent around 24.6 % in Canada in 2036, according to estimation (Turcotte M et al. 2006, Statistics Canada). Cognitive impairment and dementia is a growing health problem in elderly populations with a prevalence of 8 % for dementia and around 16 % for cognitive impairment for Canadian population (Canadian Study on Health and Aging 1994) that is a source of morbidity and reduced quality of life. These states are often preceded by a period of mild cognitive impairment (MCI) (Luck et al. 2010). The MCI state was developed in the literature and in clinical research to describe the state between cognitive changes of aging and a fully developed cerebrovascular disease (Oveisgharan et al. 2010). In the context of a prevention of cognitive impairment, the study of the MCI state and preventive interventions (such as nutritional and exercise interventions) is of clinical importance, because curative medication strategies have shown some modest effects (Purandare 2009).

Cardiovascular and metabolic risk factors and their impact on cognitive performance

Certain cardiovascular risk factors have been identified as known risk factors for decreased cognitive performance. These are the common traditional cardiovascular risks factors in heart diseases: hypertension, dyslipidemia, diabetes, obesity, heart disease, smoking, sedentarity (Purandare 2009). Epidemiological studies have shown that hypertension or/and hypercholesterolaemia in the mid-life is a risk factor for dementia in old age and confers a higher risk for dementia in young elderly (Skoog et al. 1996, Li et al. 2007, Gold et al. 2007, Kivipelto et al. 2002, Oveisgharan et al. 2010). In the same way, patients with diabetes are at a higher risk of abnormally decreased cognitive performance, and thus later of dementia, because of brain vascular damages caused by glucose dysregulation (Ott et al. 1996, Irie et al. 2008). Smoking has also been demonstrated as important risk factors for cognitive dysfunction and dementia, particularly smoking during middle age (Anstey et al. 2007). Finally, reduced physical activity during the midlife was associated with a higher risk of dementia or Alzheimer disease later in life (Rovio et al. 2005).

Cognitive function in patients with metabolic syndrome, coronary heart disease and chronic heart failure.

The metabolic syndrome (MetS) refers to a constellation of coronary heart disease (CHD) risk factors including obesity and abdominal fat distribution, disorders of glucose and lipid metabolism, and hypertension. While each of these risk factors individually has been shown to increase cardiovascular risk (Rosengren et al. 2004), MetS itself has been shown to increase cardiovascular and all cause mortality in subjects without known heart disease (Lakka et al. 2002, Isoma et al. 2001). Additionally, patients with MetS and CHD are at increased risk of death compared to CHD patients without metabolic syndrome (Nigam et al. 2006). The prevalence of obesity continues to rise at epidemic proportions in both North America and Europe, with estimation suggesting that over 50% of adults above the age of 50 in the United States have the metabolic syndrome (Ford et al. 2004). It is suggested that MetS may accelerate the physiological loss of cognitive performance with ageing, through mechanisms related to micro and macrovascular damages, insulin resistance, adiposity and inflammation (Yaffe K 2007). The MetS has been associated with accelerated cognitive decline (Yaffe et al. 2004, 2007, 2009, Liu et al. 2009, Komulainen et al. 2007, Gatto et al. 2009) in middle age and older adults, and with vascular dementia (Yaffe et al. 2004) or Alzheimer disease (Vanhanen et al. 2006) in older adults.

In CHD, the decrease in cognitive performance may be explained by a decreased cardiac output, cerebral hypoperfusion and neuronal damage. CHD is associated with increased amounts of cerebral amyloid deposits and increases the risk of dementia and Alzeimer disease (Sparks et al. 1990, Soneira et al 1996). Other studies have associated CHD with decreased cognitive performance (Aronson et al. 1990, Breteler et al. 1994, Singh Manoux et al. 2003). More precisely, a recent study has demonstrated that CHD is associated with non-amnesic mild cognitive impairment (Roberts et al. 2009). It has thus been recently proposed that CHD could be a marker of subclinical cerebrovascular disease and mild cognitive impairment (Roberts et al. 2008).

Chronic congestive heart failure (CHF) is a clinical syndrome characterized by a systemic perfusion inadequate to meet the body's metabolic demand, as a result of impaired cardiac pump function. Cognitive impairment is more important in CHF patients than in other cardiac patients (Bennet and Sauvé 2003). Loss of cognitive performance is particularly prevalent in CHF patients and 80 % of those patients will experience cognitive impairment. In CHF patients, deficits in attention, executive function, memory and psychomotor speed are common (Bennet and Sauvé 2003). Cognitive impairment in CHF patients is associated with increased mortality (Zuccala et al. 2003), reduced quality of life (Bennet and Sauvé 2003), and the degree of cognitive impairment is associated with the severity of CHF symptoms (Trojano et al. 2003). Patients with CHF have several brain abnormalities on

neuroimaging including cerebral atrophy and infarcts (Bennet and Sauvé 2003), reduced grey matter volume (Woo et al. 2003), white matter hyperintensities (Almeida et al. 2005) and alteration of cerebral metabolism (Lee et al. 1999, 2001). All those abnormalities have been related to cognitive dysfunction in patients with cardiovascular disease (Paul et al. 2005). In addition, patients with CHF have also a reduced cerebral perfusion compared with healthy controls. A study has documented a 31% lower cerebral perfusion (Gruhn et al. 2001) in patients with CHF compared with healthy controls. Other studies have showed a reduced perfusion in specific brain regions (frontal, temporal and parietal lobes) (Alves et al. 2005), and chronic cerebral hypo-perfusion contributes to cognitive dysfunction in such patients (Bennet and Sauvé 2003).

Pharmacological interventions on cognitive troubles and their vascular risk factors

Previous work has investigated the effects of different pharmacological interventions on cognitive function. Cholinesterase inhibitors have failed to improve cognitive performance or prevent the progression of dementia in older adults (Petersen et al. 2005, Feldman et al. 2007). Similarly, other agents including piracetam, cox-2 inhibitors and statins have failed to improve cognitive function (Jelic et al. 2006, Thal et al. 2005, Heart Protection study 2002). Antihypertensive treatments have showed mixed results with either some positive effects on cognition reported (Forette et al. 2002), or no effects of the treatment compared to control (Lithel et al. 2003).

Benefits of physical activity on cognitive function in humans.

Several observational studies have showed that physical activity confers a protective effect on the development of cognitive impairment. In older women, long-term physical activity was associated with a better cognitive function and a slower decline of cognition (Weuve J et al. 2004). As well, in elderly men, a lower walking distance was an independent predictor of dementia (Abbott R et al. 2004). Other prospective studies have showed that physical activity is associated with a reduced incidence of dementia (Larson et al. 2006, Podewils et al. 2005, Etgen et al. 2010) and that even in later life, participation in physical activity with a certain level intensity was associated with a better cognitive performance in men (van Gelder et al. 2004), and a lower incidence of mild cognitive impairement (MCI) in general population (Geda et al. 2010).

The data from observational studies and the clear association between physical activity and reduced risk of cognitive decline require confirmation in prospective randomized trials. Only two recent randomized trials with physical activity intervention on cognition have been performed in older adults (Lautenschlager et al. 2008, Baker et al. 2010). A 24-weeks physical activity intervention was shown to improve cognitive performance in adults with subjective impairment after 18 months of follow up (Lautenschlager et al. 2008). Baker et al. demonstrated that a six month high intensity aerobic exercise training program (Baker et al. 2010) improved cognitive function in adults with mild cognitive impairment, particularly in women. Two studies have showed that exercise training can have a positive effect on cognitive function in CHD (Carles et al. 2007) and CHF (Tanne et al. 2005). Major limitations of these two studies were their non-randomized nature, the lack of a control group, and small sample size.

The mechanisms by which physical activity may improve cognitive performance are not clear. One of the possible mechanism is environment enrichment within the context of a more physically active lifestyle (Lautenschlager et al. 2008). Animal studies have showed that environment enrichment leads to an increased physical activity, increasing brain plasticity via synaptogenesis and neurogenesis (Kronemberg et al. 2006, Uda et al. 2006). In humans, a randomized trial showed the positive effects of a 6 months aerobic training program on task related activity in brain attentional control areas, suggesting improved synaptogenesis and cholinergic effect (Colcombe et al. 2004).

A second potential mechanism is an improvement of cerebral vascular function and brain perfusion via chronic exercise. Animal studies showed that exercise training improves angiogenesis, brain perfusion and cerebral vascular function (Swain et al. 2003). In humans, Colcombe et al. demonstrated that aerobic exercise training is associated with increased blood perfusion of the brain regions that modulate attention in older adults (Colcombe et al. 2004). Other studies have shown that high aerobic fitness has a protective effect on the brain tissue loss during aging (Colcombe et al. 2003), and results in higher hippocampal brain volume and memory function (Erickson et al. 2009). Finally, 6 months of aerobic exercise training increased brain volume in prefrontal and temporal regions in older adults (Colcombe et al. 2006).

Acute exercise and cerebral oxygenation in healthy subjects and cardiac patients.

The cerebral NIRS technique (Near InfraRed Spectroscopy) has been used to document brain oxygenation in healthy subjects during incremental maximal exercise tests. These studies primarily explored the prefrontal cortex zone (Perrey S 2008) during exercise. In healthy subjects, brain oxygenation (measured with O2 haemoglobin signal) has been shown to increase during light to moderate intensity exercise but decreases near maximal exercise intensity (Bhambhani et al. 2007, Subudhi et al. 2007, 2008, Thomas et al. 2008, Peltonen et al. 2009). Furthermore, there is also a gradual increase of brain blood volume (measured with total haemoglobin signal) during incremental exercise in healthy subjects. Thus, the relationship between cerebral oxygenation and exercise intensity follows an inverted U-shape (Gonzalez-Alonso et al. 2004; Bhambhani et al. 2007; Rupp et al 2008). In certain subjects with cardiovascular disease, this inverted U-shape curve is absent, although there still exists an exercise intensity treshold above which cerebral oxygenation suddenly decreases (Koike, Itoh et al. 2004; Koike et al. 2006; Koike et al. 2008). In patients with heart failure or valvular heart disease, Koike et al. showed that there was a less important increase in cerebral O2 Hb compared to healthy controls during incremental exercise, with 45 % patients demonstrating a paradoxical decrease of O2 Hb compared to healthy controls (Koike et al. 2004 a et b). In these same studies, the increase in cerebral O2 Hb correlated with peak VO2 and left ventricular ejection fraction in both healthy subjects and cardiac patients, reflecting a link between cardiac output and cerebral O2 Hb increase (Koike et al. 2004 a et b, Nagayama et al. 2007). In subjects with severe CHF, a marked drop of cerebral O2 Hb can be critical and lead to a loss of consciousness (Koike et al. 2006). In CHD patients, cerebral O2 Hb during exercise has been shown to be related to cerebrovascular disease as revealed by MRI flair score (Nagayama et al. 2007). CHD patients with more advanced cerebrovascular disease had a lower cerebral O2 Hb during exercise, peak VO2 and left ventricular ejection fraction (Nagayama et al. 2007). More recently, a decrease of cerebral O2 Hb during exercise in patients with CHD was found to be an independent predictor of cardiovascular events (Koike et al. 2008).

Effects of exercise training on physical and cognitive performance

The latest recommandations of the American Heart Association on exercise prescription in patients with metabolic syndrome and CHD suggest that exercise intensity should lie between 50% and 80% of maximal aerobic power (MAP) (Balady et al. 2007). However, recent studies about high-intensity intermittent exercise (HIIE) show promising results. HIIE involves repeated 30 to 300-second bouts of aerobic exercise at a higher intensity, interspersed by recovery periods of equal or shorter duration (Daniels et al. 1984). HIIE has largely been used by elite athletes for training purposes since then (Billat, 2001). Recent data indicates that HIIE permits individuals to achieve a higher power output compared to standard moderate-intensity continuous exercise(MICE). Maintaining a higher power output is beneficial to increasing both maximal oxygen uptake (VO2max por peak VO2) and the anaerobic treshold, therefore postponing apparition of fatigue during exercise. Studies have compared

MICE to HIIE and have reported a greater increase in cardiac output and stroke volume following HIIE training in healthy subjects and athletes (Laursen et al. 2002; Daussin et al. 2007; Daussin et al. 2008). These results have also been reported in patients with metabolic syndrom (Tjonna et al. 2008) and in elderly subjects with CHD (Meyer et al. 1990; Rognmo et al. 2004) and stable CHF (Wisloff et al. 2007). These studies demonstrate that the practice of HIIE is safe and even beneficial to slow down the drop in cardiac output associated with ageing. Morever, we recently developed a specific HIIE training modality for CHD patients, each training session consisting of two sets of 10 minutes composed of repeated bouts of 15 s at 100% of MAP interspersed by 15 s of passive recovery. This modality represents the best compromise between safety, time spent at a high level of VO2peak and perceived exertion (Guiraut et al. 2010 a; b).

While previous studies have shown interesting results comparing physical fitness and cognition in elders, it is unknown wether a specific aerobic training modality can lead to a greater improvement in cognitive performance in older adults. Given its greater impact on cardiac output and stroke volume, it can be hypothesized that HIIE induces larger increases in cerebral blood flow and perfusion relative to MICE. To our knowledge, this hypothesis has never been studied in elders, nor in cardiac patients.

MÉTHODES ET MESURES À EFFECTUER

1. Description de la population :

STUDY 1:

A total of 80 patients with different cardiovascular conditions will be recruited. Healthy control subjects without evidence of CHD or no cardiovascular risk factors will be accepted. As well, subjects without evidence of CHD but with several cardiovascular risk factors (MetS patients) will be recruited and finally, patients with documented CHD and CHF will be also accepted.

- 20 healthy controls subjects will be recruited.
- 20 patients with MetS (and no-CHD) will be recruited.
- 20 patients with CHD will be recruited.
- 20 patients with CHF will be recruited.

STUDY 2:

Study 2 will be proposed to patients with MetS, CHD or CHF who accepted to be enrolled in study 1

- 20 patients with MetS (and no-CHD) will be recruited.
- 20 patients with CHD will be recruited.
- 20 patients with CHF will be recruited.

2. Critères d'inclusion :

Healthy subjects : with no MetS and no-documented CHD, both males and females, aged>18 years will be included in the study, should they provide written informed consent and have a sufficient initial physical and intellectual capacities allowing an independent daily living.

Patients with MetS and no-documented CHD, both males and females, aged > 18 years will be included in the study, should they provide written informed consent and have a sufficient initial physical and intellectual capacities allowing an independent daily living. MetS will be defined according to recent updated criteria (Alberti et al. 2005):

presence of at least three of five criteria, namely abdominal obesity (waist circumference cut-off depending on the recently published ethnic-based variations (Genest et al. 2009)), triglycerides > 1.70 mmol/l, decreased HDL-cholesterol (< 1.0 mmol/l in men and < 1.3 mmol/l in women), systolic blood pressure > 130 mmHg or diastolic blood pressure > 85 mmHg, and FPG > 5.6 mmol/l.

CHD patients, both males and females, aged > 18 years will be included in the study, should they provide written informed consent and have a sufficient initial physical and intellectual capacities allowing an independent daily living. Moreover, they must have documented CHD (prior myocardial infarction, prior coronary angiography or angioplasty, or documented myocardial ischemia on myocardial scintigraphy).

Patients with documented stable chronic heart failure will be recruited if they show the following inclusion criteria:
- ≥18 years
- Left ventricular ejection fraction (LVEF) <40% (measured within 6 months of their enrolment by MUGA Scan, echo or radiological ventriculography)
- NYHA functional class I-III
- Optimal therapy at stable doses including a beta-blocker and an ACE inhibitor or ARA for at least 6 weeks prior to investigation (unless documented rationale for variation).
- Able to perform an symptom limited exercise test.
- Capacity and willingness to sign the informed consent form.

STUDY 2:

Twenty patients with MetS, 20 patientswith CHD will be included. Patients with stable congestive CHF will also be included. Inclusion criteria will be the same as described above.

3. Critères d'exclusion :

For healthy subjects:

- lack of expressed written consent
- metabolic syndrome
- coronary heart disease
- chronic systolic heart failure
- resting left ventricular ejection fraction < 40 %
- symptomatic aortic stenosis
- chronic atrial fibrillation
- malignant exertional arrhythmias
- non-cardiopulmonary limitation to exercise (e.g: arthritis or claudication)
- severe exercise intolerance.

For patients with MetS:

- lack of expressed written consent
- coronary heart disease
- chronic systolic heart failure
- resting left ventricular ejection fraction < 40 %
- symptomatic aortic stenosis
- chronic atrial fibrillation
- malignant exertional arrhythmias
- non-cardiopulmonary limitation to exercise (e.g: arthritis or claudication)
- severe exercise intolerance.

For patients with CHD

- lack of expressed written consent
- recent acute coronary event (< 3 months)
- chronic systolic heart failure
- resting left ventricular ejection fraction < 40 %
- symptomatic aortic stenosis
- severe non-revascularizable coronary disease including left main coronary stenosis
- patient awaiting coronary artery bypass surgery
- chronic atrial fibrillation
- presence of permanent ventricular pacemaker
- malignant exertional arrhythmias
- non-cardiopulmonary limitation to exercise (e.g: arthritis or claudication)
- severe exercise intolerance.

For CHF patients:

- Any relative or absolute contraindications to exercise training among patients with stable chronic heart failure according to current recommendations (Working Group on Cardiac Rehabilitation 2001)
- Fixed-rate pacemaker or ICD devices with heart rate limits set lower than the exercise training target heart rate.
- Major cardiovascular event of procedure within the 3 months preceding enrolment in the study.
- Atrial fibrillation
- Heart failure secondary to significant uncorrected primary valvular disease (except for mitral regurgitation secondary to LV dysfunction)
- Heart failure secondary to congenital heart disease or obstructive cardiomyopathy.

4. Plan de recherche (protocole) :

STUDY 1:

All the subjects will undergo a total of 4 visits in the ÉPIC Centre to complete all the tests and measurement required for this project.

First Visit: baseline evaluation and vascular reactivity evaluation

After filling the informed consent form, patients will undergo a measurement of their body composition with a bioelectrical impedance analysis (Tanita BC 418, Japan) and their height. Medical history will be recorded from patient's file. A complete standard blood analysis will be performed in a fasting state for the following parameters:Total cholesterol, HDL, LDL cholesterol, triglycerides, insulin, glycemia, glycated haemoglobin HbA1c, C-reactive protein (CRP), Angiopoietin-like 2, and C-terminal BNP for CHF patients.
Vascular reactivity will be assess at the level of the forearm and the brain using the NIRS technology. At the brain level, measures will be performed twice so as to study reproducibility.

Second Visit: cognitive function evaluation (see details in "Mesures à effectuer").

Third visit: maximal cardiopulmonary exercise testing
Patients will perform a maximal cardiopulmonary exercise testing (CPET) on bicycle ergometer (Ergoline 800S, Bitz, Germany) using an incremental protocol with gas exchange analysis. Additionally, cardiac output (cardiac bioimpedance) and brain NIRS signals will be measured during maximal exercise. See measurement for further methodological details.

Fourth visit: cognitive test during sub-maximal exercise.
After a 2 minutes warm up at 20 Watts , the patient will perform a computerized Stroop tes twhile pedaling under and over the mechanical power corresponding to his cerebral oxygenation treshold. See measurement for further methodological details.

STUDY 2:

Patients will be randomized to a high intensity interval training (HIIIE) or a moderate isocaloric continous training program (MICE) according to the model developed in our laboratory (Guiraud et al. 2010 a, Guiraud et al 2010 b).

Patients will train at the Centre ÉPIC for a period of 12 weeks, 3 to 5 times per week, with a session duration of 35 min for the MICE and 29 min for the HIIE session. For CHF patients, sessions will last 16 minutes in the MICE group, and 22 minutes in the HIEE group

At the end of the training period, they will undergo 4 visits to perform the same tests as the visit 1; 2; 3; and 4 of study 1.

5. Mesures à effectuer :

Blood analysis

It will be performed by a nurse at the centre EPIC in a fasting state. It will include the following parameters: Total cholesterol, HDL and LDL cholesterol, triglycerides, insulin, glycemia, glycated haemoglobin HbA1c, C-reactive protein (CRP). For CHF patients, C-terminal BNP dosage will also be performed. For patients included in Study 2, Angiopoietin-like 2 dosage will also be performed at visit 1 of study 1 and visit 1 of study 2. Angiopoietin-like 2 is an inflammatory circulating protein. Dr Thorin's team demonstrated that it is also an important vasodilator factor, by producing GMPc via the activation of the NO-dependant guanylate cyclase. This factor has been identified from endothelial cells of vessels in patients undergoing a coronary bypass, and probably plays a role in the endothelial adaptations during a training period.

Vascular reactivity at the forearm level:

Examinations will be performed while patients are still resting in the supine position. NIRS measurements will be carried out on top of the flexor digitorum superficialis muscle (FDS) with an interoptode distance of 45 mm. In order to prevent variations in placement of the optodes and to avoid operator errors, the angle and place of the optodes will be kept constant during the test using a support that will be attached to the skin with adhesive stickers. Data will be sampled at 10 Hz, displayed realtime and stored on disk for off-line analysis (Van Beekvelt et al. 2001).

A pneumatic blood pressure cuff will be positioned above the elbow. The NIRS signals will be sampled at rest during 2 minutes before, during venous and arterial occlusion, and after cuff inflations (3 minutes post cuff deflation).
After the rest period, the cuff will be inflated to 50 mm Hg for 1 minute. Then the cuff will be then released, and a second venous occlusion will be performed 1 minute later (Van Beekvelt et al. 2001). FBF will be calculated from NIRS data (FBFNIRS) by evaluating the linear increase in [tHb] within the first seconds of the venous occlusion (Van Beekvelt et al. 2001). Concentration changes of [tHb] will be expressed in micromolars per second and were converted to milliliters blood per 100 milliliters tissue per minute by using the individual Hb concentration that was obtained from the blood samples. The molecular weight of Hb (64.458 g/mol) and the molecular ratio between Hb and O2 (1:4) will be taken into account.
After 2 minutes of recovery, arterial occlusion will be performed during 5 minutes and signal will be measured during 5 minutes after arterial occlusion.
The following NIRS parameters will be used during NIRS arterial occlusion (Kragelj et al. 2001):

1- HMax, maximal hyperaemic response in %, maximal change of the signal after the release of the cuff, expressed as the percentage of the signal change than is higher than the pre occlusion baseline values.

2- HR, total hyperaemic response in %, maximal change of the Total Hb (THb) signal after the release of the cuff, expressed as the percentage of the signal change during arterial occlusion.

3- VO2, oxygen consumption, calculated from the gradient of the HbO2 signal during arterial occlusion and converted to ml/min as described elsewhere (Cheatle et al. 1991)

4- TR, time of recovery, the time interval after release of the cuff until the initial pre occlusion values of HbO2, Hb signals are reached.

5- TM, time to peak value, the time interval between the release of the cuff and the moment of the maximum hyperaemic responses for Total Hb signal.

6- TMO2, time to peak value, the time intervall between the release of the cuff and the moment of the maximum hyperaemic responses for HbO2 signal.

7-TB, time to baseline, the time interval between the release of the cuff and the return to pre occlusion baseline value.

At the brain level vascular reactivity will be assessed with the breath holding test (BH).

Changes in the concentrations of O2Hb, CO2Hb and total HHb will be measured by NIRS (Oxymon, Artinis Medical Systems, Nijmegen, the Netherlands). The emitting probe of the NIRS equipment will be placed on the left frontal side of the subjects, 2 cm beside the midline and about 3 cm above the supraorbital ridge (Molinari et al. 2006). We will chose this positioning in order to avoid the sinuses and to place the probes on a poorly perfused and very thin skin layer. Breath holding is supposed to induce a perturbation in cerebral cortex that is systemic and not regional or localized; hence the frontal lobe is a suitable location also for the absence of hairs. According to previous studies and theoretical models already developed, a differential pathlength factor equal to 5.97 and an interoptode distance of 45 mm will be set (Molinari et al. 2006).
Patients will rest during 5 minutes in the supine position before installation of NIRS probe on the brain. Baseline NIRS signals will be measured during 3 minutes (Molinari et al. 2006, Safonova et al 2005).
Then, breath holding test will be performed. Subjects will be asked to hold their breath at the end of normal expiration for as long as they felt comfortable (15–30 s) without forcing themselves (to avoid the Valsalva maneuver). After the resumption of breathing and the recovery of the baseline level of SaO2, subject will be asked to repeat breath holding at the end of a full inspiration with a Valsalva maneuver as long as they felt comfortable (15–30 s). This sequence will be repeated one time, then NIRS signals will be measured 3 minutes after the 2 breath holding test.

Cognitive Function evaluation

A set of paper-and-pencil and computerized tests involving short-term memory, long-term memory, attention, and executive functions will be administered to the patients.
The Mini Mental State Examination (MMSE, Folstein, Folstein, & McHugh, 1975; cut-off score < 26/30) and the Geriatric Depression Scale (GDS, Yesavage et al., 1983; cut-off score > 10/30) will first be used to exclude participants suffering from dementia or depression. The test battery includes clinical tests that are widely used in clinic and that are validated (Lezak, 2004) along with experimental cognitive tasks that offer greater sensitivity and power. This battery has been developped by Pr Bherer, specialized in cognitive evaluation and ageing. The entire set is usually performed within 1 hour. It will be administered by an examiner experienced in neuropsychological test. The tests used will be the following:

1. Digit Span (Forward and Backward): starting from 2 digits, participant is asked to repeat the digits in correct serial order (forward) until he reaches the longest span without error. Same procedure for the backward span.
2. Digit Symbol Substitution Test: the participant must associate symbols to numbers (1 to 9) by referring to a response key consisting of rectangles containing a number in the top part and a symbol in the bottom part.
3. Trail making test part A and B: in part A, the participant must link up numbers (from 1 to 25) in a continuous line as fast as possible. In Part B, the participant must alternate between letters in alphabetical order and numbers in ascending order (1-A-2-B-3-C, etc.) as fast as possible.
4. D-KEFS Color-Word Interference Test: is based on the Stroop procedure. The test has two baseline conditions that measure key components skills of higher level tasks: basic naming of color patches and basic reading of color-words printed in black ink. A third condition: inhibition is the traditional Stroop task, for which the participant must inhibit reading the words in order to name the dissonant ink colors in which those words are printed. The D-KEFS test included a fourth condition inbition/swithching. For this condition, the participant is asked to switch back and forth between naming the dissonant ink colors and thus reading the words. This condition is

thus a means of evaluating both inhibition and cognitive flexibility.

5. Rey Auditory Learning Test: This test consists of five presentations with recalls of a 15-word list, and one presentation and recall of a second 15-word list followed by a sixth recall trial of the first list. Thirty minutes following this learning phase a delayed recall of the first list is performed followed by a recognition test.
6. Computerised dual task: in the dual task, the participant first executed two visual tasks separately (1) identification of an arrow pointing right or left and (2) discrimination between a red and a green square. In the dual task condition the participant must perform one or the other visual task or both tasks at the same time.

Maximal cardiopulmonary exercise test

Ergometer

Exercise testing will be performed on an electro-mechanically braked bicycle ergometer (Ergoline 800S, Bitz, Germany). Cycling position, which is known to affect energy expenditure, will be standardized by adopting a top bar position. Saddle height will be adjusted according to the participant's inseam leg length, as measured from the point of the pubic symphysis palpation to the floor. Toe-clips will be used and participants will be instructed to stay seated during the test. Subjects will have to maintain a constant pedal cadency between 60 and 80 revolutions per minute

Exercise protocol and gas exchanges measurement

The test will begin immediately after a 3-min warm-up phase at 20 W. Depending on cardiovascular status of the patient, initial workload will be individualized and increased by 5 to 15 W every minute until exhaustion. Strong verbal encouragement will be given throughout the test. The power of the last completed stage will be considered as the maximal aerobic power (MAP). Oxygen uptake (VO2) will be measured continuously on a breath-by-breath basis using an automated cardiopulmonary exercise system (Oxycon Pro, Jaegger, Germany). Gas analyzers will be calibrated before each test, using a gas mixture of known concentration (15% O2 and 5% CO2) and ambient air. Participants will breath through a facemask connected to a turbine with low resistance. The turbine will be calibrated before each test using a 3-l syringe at several flow rates. Electrocardiographic activity will be monitored continuously using a ECG (Marquette, Missouri) and blood pressure was measured manually every 2 min using a sphygmomanometer. Peak VO2 and peak heart rate will be defined as the mean oxygen uptake and heart rate values during the last 15 s of exercise. The ventilatory thresholds will be determined using the method of Beaver et al.

Cardiac bioimpedance

Cardiac bioimpedance (Physioflow, Enduro model, Manatec, France) will be used to measure central hemodynamic modifications during exercise. Cardiac output, stroke volume, heart rate, ventricular ejection time, end diastolic volume and systemic vascular resistance will be measured with this device. The theoretical basis for this technique and its application and validity at rest and for exercise testing have been previously described (Charloux et al. 2000, Richard et al. 2001). The physioflow device measures impedance changes (dZ) in responses to a small-administered electrical current. Two sets of 2 electrodes (Ag/AgCl, Skintact, F-TB model, Austria), one electrode transmitting and the other sensing, respectively, will be applied above the supra- clavicular fossa at the left base of the neck and along the xiphoid. A further set of two electrodes will be used to monitor a single ECG (CM5 position). Electrodes will be taped with transparent dressings (Tegaderm, 3M, USA). With this impedance device, a first evaluation of stroke volume index (SVi) will be calculated during a calibration procedure based on 30 consecutive heartbeats recorded in the resting condition (SVical). This evaluation keeps the largest impedance variation during the systole (Zmax _ Zmin) and the largest rate of variation of the impedance signal (dZ/dtmax, called the contractility index). The SVi calculation also depends on the thoracic flow inversion time (TFIT, in m•s_1) measured on the first mathematical derivative of the impedance signal. TFIT is the time interval between the first zero value after the beginning of the cardiac cycle (beginning of the ECG's QRS complex) and the first nadir after the peak of the ejection velocity (dZ/dtmax). Afterward, TFIT is weighted using a specific algorithm. During the data acquisition phase, the variation of parameters will be analyzed and compared with those obtained during the calibration procedure. So, the stroke volume (SV) is the product between the electrical physical volume of thorax (VEPT), the ventricular ejection time (VET), and the maximum rate of the impedance change during the systolic upstroke [(dZ/t)max] divided by the basal thoracic impedance (Z0). For this experiment, bioimpedance variables will be measured continuously during each test with a 15 consecutive heartbeats average recorded. Q calculation by the device is based on the following formula: Q= HR × SVi × BSA, where Q is expressed in liters per minute, HR is the heart rate based on the R-R interval measurement, determined on the ECG first derivative, dECG/dt, which provides a more stable signal than the ECG signal itself.

Cerebral oxygenation and blood flow measurement with the NIRS during exercise

The emitting probe of the NIRS equipment will be placed on the left frontal side of the subjects, 2 cm beside the midline and about 3 cm above the supraorbital ridge (Bhambhani et al. 2007). We will choose this positioning in order to avoid the sinuses and to place the probes on a poorly perfused and very thin skin layer. The probe will be secured with a tensor bandage wrapped around the forehead, taking sufficient care to ensure that there will be no loss of background light. The metabolic headgear will be placed over the probe so as to prevent it from moving. The receiving sensor will be fixed laterally to the emitter at a distance of about 5 cm. According to previous studies and theoretical models already developed, a differential pathlength factor equal to 5.97 will be set (Molinari et al. 2006). The NIRS brain signals will sampled at a frequency of 10 Hz and will be measured during rest period, exercise and recovery. The Cerebral Oxygenation Treshold (COT) will be determined as the level of exercise above which cerebral oxygenation declines.

Submaximal Exercise test:
After a 2 minutes warm up at 20 Watts, the patient will perform the following sequence:
-A first 3 minutes period at an intensity 10% lower than the power output corresponding to the COT, determined during the maximal exercise test performed during visit 3. A computerized Stroop test will be performed during the 3rd minute.
- Then 2 minutes of passive recovery will follow
-The patient will then perform a new 2 minutes period pedalling at 20 Watts, immediately followed by a 3 minutes period at an intensity 10% over the COT. A new computerized Stroop test will be performed at the 3rd minute.
During this test, electrocardiographic activity will be monitored continuously using a ECG (Marquette, Missouri) and blood pressure will be measured manually every 2 min using a sphygmomanometer.

Exercise training interventions

Patients will train at the centre EPIC for a period of 12 weeks, 3 to 5 times per week, with a session duration of 35 minute for the MICE group and 29 minutes for the HIIE group. For CHF patients, sessions will last 16 minutes in the MICE group and 22 minutes in the HIEE group. All sessions will be perfor;ed on a bicycle ergormeter, and will be supervised by an experienced kinesiologist and a doctor.

Moderate Intensity Continuous Exercise (MICE) session
This exercise session will be based on the recommendations of the American Heart Association on exercise prescription in cardiac rehabilitation (Balady et al. 2007), suggesting that exercise intensity should lie between 50% and 80% of maximal aerobic power (MAP). We opted for an intensity of 70% of MAP. Duration will be adjusted to match total energy expenditure of the HIIE, according to the previous methodology method (Guiraud et al. 2010 b). A total duration of 28.7 minutes will be employed in coronary artery disease patients and patients with metabolic syndrom; and 16 minutes in chronic heart failure patients.

High-intensity interval exercise (HIIE) session
This HIIE session will be based on a previous study conducted in our laboratory that compared physiological, psychological and electrocardiological tolerance of four different single bouts of HIIE in coronary patients (Guiraud et al. 2010). The selected HIIE session represented the best compromise between safety, time spent at a high level of VO2peak and psychological adherence. This HIIE session consists of a 10-min warm-up at 50% of MAP, followed by two sets of 10 min composed of repeated bouts of 15 s at 100% of MAP interspersed by 15 s of passive recovery. Four minutes of passive recovery were allowed between the two sets, as well as a 5-min cool-down after the last 15-s exercise bout. A total duration of 35 minutes will be employed for this session for coronary artery disease patients, and 22 minutes for chronic heart failure patients (Guiraut et al. 2010 b).

ASPECTS STATISTIQUES

1. Critères d'évaluation :

2. Primaires :

Volet 1: le critère primaire sera la corrélation entre performance cognitive et oxygénation cérébrale à l'effort

Volet 2: Amélioration de la performance cognitive avant-après entraînement physique de 12 semaines

3. Secondaires :

VOLET 1:

- Corrélations entre performance cognitive et VO2 max, débit cardiaque,et réactivité cérébro-vasculaire.
- Reproductibilité du Breath Holding Index dans cette population.
- Corrélations entre résultats du BHI et la réactivité vasculaire mesurée à l'avant bras par les méthodes d'occlusion veineuse et artérielle.

VOLET 2:
- Comparaison des profils d'oxygénation cérébrale au cours d'une séance ECIM et une séance EIHI.
- Comparaison des améliorations des capacités cognitives et de VO2 pic, du débit cardiaque, de l'oxygénation cérébrale, et réactivité cérébro-vasculaire, entre les 2 groupes

4. Calcul de la taille d'échantillonnage :

Volet 1

For a multiple linear regression model on predicting Cognitive Function which aleady includes 5 covariates with a squared multiple correlation R^2 of 0.50, a sample size of 80 will have 80% power to detect at alpha=0.05 an increase in R^2 of 0,046 due to including the factor of interest (ex: HbO^2). The number of covariates (ex: from 1 to 15) has only a very small effect on the results.

Volet 2

For a multiple linear regression model on predicting Cognitive Function which aleady includes 5 covariates with a squared multiple correlation R^2 of 0.50, a sample size of 60 will have 80% power to detect at alpha=0.05 an increase in R^2 of 0,060 due to including the factor of interest (ex: HbO^2). The number of covariates (ex: from 1 to 15) has only a very small effect on the results.

5. Analyses statistiques :

To study the relationship between factors (ex: Age, Sex, HDL, VO^2, Cardiac Output, HbO^2, exercise, training, etc.) and the Cognitive Function (CF), a multiple linear regression will be used. A forward stepwise approach will be used to select statistically significant risk factors. The order of inclusion in the multiple linear regression will be based on hypotheses to be tested. Goodness of fit statistics will include studentized deleted residuals, leverage values,

Cook's distances and variance inflation factors. If necessary, remedial measures will be used such as weighted least squares, transformations or nonparametric regressions.
The same approach will be used when using as dependant variables others enpoints such as HbO^2, Cardiac Output, VO^2 max, etc.

INFORMATIONS SUR LES MÉDICAMENTS

☒ **NON APPLICABLE**

EXPÉRIMENTATION OU À USAGE EXCEPTIONNEL

(Cette section doit être complétée en entier, si (un) médicament à évaluer n'est pas commercialisé au Canada)

1. Nom du médicament :

2. Actions principales et caractéristiques :

3. Présentation :

4. Posologie :

5. Effets secondaires (par ordre de fréquence) :

6. Paramètres (signes vitaux) et laboratoires à surveiller :

INFORMATIONS SUR LA NOUVELLE TECHNOLOGIE OU SUR L'INSTRUMENT MÉDICAL

☒ **NON APPLICABLE**

1. INFORMATIONS TECHNIQUES

2. Fournir photos ou images le cas échéant

BIBLIOGRAPHIE

Abbott RD, White LR, Ross GW, Masaki KH, Curb JD, Petrovitch H. Walking and dementia in physically capable elderly men. JAMA. 2004 Sep 22;292(12):1447-53.

Alberti KG, Zimmet P, Shaw J; IDF Epidemiology Task Force Consensus Group. The metabolic syndrome – a new worldwide definition. Lancet 2005;366:1059-62.

Amundsen BH, Rognmo Ø, Hatlen-Rebhan G, Slørdahl SA. High-intensity aerobic exercise improves diastolic function in coronary artery disease. Scand Cardiovasc J. 2008; 42(2):110-7.

Almeida JR, Alves TC, Wajngarten M, Rays J, Castro CC, Cordeiro Q, Telles RM, Fraguas RJ, Busatto GF. Late-life depression, heart failure and frontal white matter hyperintensity: a structural magnetic resonance imaging study. Braz J Med Biol Res. 2005; 38(3):431-6.

Alves TC, Rays J, Fráguas R Jr, Wajngarten M, Meneghetti JC, Prando S, Busatto GF. Localized cerebral blood flow reductions in patients with heart failure: a study using 99mTc-HMPAO SPECT. J Neuroimaging. 2005;15(2):150-6.

Anstey KJ, von Sanden C, Salim A, O'Kearney R. Smoking as a risk factor for dementia and cognitive decline: a meta-analysis of prospective studies. Am J Epidemiol. 2007 15; 166(4):367-78.

Aronson MK, Ooi WL, Morgenstern H, Hafner A, Masur D, Crystal H, Frishman WH, Fisher D, Katzman R.Women, myocardial infarction, and dementia in the very old. Neurology. 1990; 40(7):1102-6.

Baker LD, Frank LL, Foster-Schubert K, Green PS, Wilkinson CW, McTiernan A, Plymate SR, Fishel MA, Watson GS, Cholerton BA, Duncan GE, Mehta PD, Craft S. Effects of aerobic exercise on mild cognitive impairment: a controlled trial. Arch Neurol. 2010; 67(1):71-9.

Balady GJ, Williams MA, Ades PA, et al. Core components of cardiac rehabilitation/secondary prevention programs: 2007 update: a scientific statement from the American Heart Association Exercise, Cardiac Rehabilitation, and Prevention Committee, the Council on Clinical Cardiology; the Councils on Cardiovascular Nursing, Epidemiology and Prevention, and Nutrition, Physical Activity, and Metabolism; and the American Association of Cardiovascular and Pulmonary Rehabilitation. Circulation. 2007;115(20):2675-2682.

Beaver WL, Wasserman K, Whipp BJ. A new method for detecting anaerobic threshold by gas exchange. J Appl Physiol 1986; 60:2020–2027.

Bhambhani Y, Malik R, Mookerjee S. Cerebral oxygenation declines at exercise intensities above the respiratory compensation threshold. Respir Physiol Neurobiol. 2007 14; 156(2):196-202.

Bennett SJ, Sauvé MJ. Cognitive deficits in patients with heart failure: a review of the literature. J Cardiovasc Nurs. 2003;18(3):219-42

Billat VL, Slawinksi J, Bocquet V, Chassaing P, Demarle A, Koralsztein JP. Very short (15s-15s) interval-training around the critical velocity allows middle-aged runners to maintain VO2 max for 14 minutes. Int J Sports Med. 2001 Apr;22(3):201-8.

Breteler MM, Claus JJ, Grobbee DE, Hofman A. Cardiovascular disease and distribution of cognitive function in elderly people: the Rotterdam Study. BMJ. 1994 18; 308 (6944):1604-8.

Canadian study of health and aging: study methods and prevalence of dementia. CMAJ. 1994 15; 150(6):899-913.

Carles S Jr, Curnier D, Pathak A, Roncalli J, Bousquet M, Garcia JL, Galinier M, Senard JM. Effects of short-term exercise

and exercise training on cognitive function among patients with cardiac disease. J Cardiopulm Rehabil Prev. 2007; 27(6):395-9.

Charloux A, Lonsdorfer-Wolf E, Richard R, Lampert E, Oswald-Mammosser M, Mettauer B, Geny B, Lonsdorfer J. A new impedance cardiograph device for the non-invasive evaluation of cardiac output at rest and during exercise: comparison with the "direct" Fick method. Eur J Appl Physiol. 2000; 82(4):313-20.

Cheatle TR, Potter LA, Cope M, Delpy DT, Coleridge Smith PD, Scurr JH. Near-infrared spectroscopy in peripheral vascular disease. Br J Surg. 1991 Apr;78(4):405-8.

Colcombe SJ, Erickson KI, Raz N, Webb AG, Cohen NJ, McAuley E, Kramer AF. Aerobic fitness reduces brain tissue loss in aging humans. J Gerontol A Biol Sci Med Sci. 2003; 58(2):176-80.

Colcombe SJ, Kramer AF, Erickson KI, Scalf P, McAuley E, Cohen NJ, Webb A, Jerome GJ, Marquez DX, Elavsky S. Cardiovascular fitness, cortical plasticity, and aging. Proc Natl Acad Sci U S A. 2004 Mar 2;101(9):3316-21. Epub 2004 Feb 20.

Colcombe SJ, Erickson KI, Scalf PE, Kim JS, Prakash R, McAuley E, Elavsky S, Marquez DX, Hu L, Kramer AF. Aerobic exercise training increases brain volume in aging humans. J Gerontol A Biol Sci Med Sci. 2006 Nov;61(11):1166-70.

Daniels J, Scardina N. Interval training and performance. Sports Med. 1984 Jul-Aug;1(4):327-34.

Daussin FN, Ponsot E, Dufour SP, Lonsdorfer-Wolf E, Doutreleau S, Geny B, Piquard F, Richard R. Improvement of VO2max by cardiac output and oxygen extraction adaptation during intermittent versus continuous endurance training. Eur J Appl Physiol. 2007; 101(3): 377-83.

Daussin FN, Zoll J, Dufour SP, Ponsot E, Lonsdorfer-Wolf E, Doutreleau S, Mettauer B, Piquard F, Geny B, Richard R. Effect of interval versus continuous training on cardiorespiratory and mitochondrial functions: relationship to aerobic performance improvements in sedentary subjects. Am J Physiol Regul Integr Comp Physiol. 2008; 295(1):R264-72.

Etgen T, Sander D, Huntgeburth U, Poppert H, Förstl H, Bickel H. Physical activity and incident cognitive impairment in elderly persons: the INVADE study. Arch Intern Med. 2010 25;170 (2):186-93.

Erickson KI, Prakash RS, Voss MW, Chaddock L, Hu L, Morris KS, White SM, Wójcicki TR, McAuley E, Kramer AF. Aerobic fitness is associated with hippocampal volume in elderly humans. Hippocampus. 2009 Oct;19(10):1030-9.

Feldman HH, Ferris S, Winblad B, Sfikas N, Mancione L, He Y, Tekin S, Burns A, Cummings J, del Ser T, Inzitari D, Orgogozo JM, Sauer H, Scheltens P, Scarpini E, Herrmann N, Farlow M, Potkin S, Charles HC, Fox NC, Lane R. Effect of rivastigmine on delay to diagnosis of Alzheimer's disease from mild cognitive impairment: the InDDEx study. Lancet Neurol. 2007 Jun;6(6):501-12.

Folstein MF, Folstein SE, McHugh PR. "Mini-mental state". A practical method for grading the cognitive state of patients for the clinician. J Psychiatr Res. 1975 Nov;12(3):189-98.

Ford ES, Giles WH, Mokdad AH. Increasing prevalence of the metabolic syndrome among U.S. Adults. Diabetes Care 2004; 27:2444-9.

Forette F, Seux ML, Staessen JA, Thijs L, Babarskiene MR, Babeanu S, Bossini A, Fagard R, Gil-Extremera B, Laks T, Kobalava Z, Sarti C, Tuomilehto J, Vanhanen H, Webster J, Yodfat Y, Birkenhäger WH; Systolic Hypertension in Europe Investigators. The prevention of dementia with antihypertensive treatment: new evidence from the Systolic Hypertension in Europe (Syst-Eur) study. Arch Intern Med. 2002 Oct 14;162(18):2046-52

Gatto NM, Henderson VW, St John JA, McCleary C, Hodis HN, Mack WJ. Metabolic syndrome and cognitive function in healthy middle-aged and older adults without diabetes. Neuropsychol Dev Cogn B Aging Neuropsychol Cogn. 2008;15(5):627-41.

Geda YE, Roberts RO, Knopman DS, Christianson TJ, Pankratz VS, Ivnik RJ, Boeve BF, Tangalos EG, Petersen RC, Rocca WA. Physical exercise, aging, and mild cognitive impairment: a population-based study. Arch Neurol. 2010; 67(1):80-

6.

Genest J, McPherson R, Frohlich J, Anderson T, Campbell N, Carpentier A, Couture P, Dufour R, Fodor G, Francis GA, Grover S, Gupta M, Hegele RA, Lau DC, Leiter L, Lewis GF, Lonn E, Mancini GB, Ng D, Pearson GJ, Sniderman A, Stone JA, Ur E. 2009 Canadian Cardiovascular Society/Canadian guidelines for the diagnosis and treatment of dyslipidemia and prevention of cardiovascular disease in the adult - 2009 recommendations. Can J Cardiol. 2009 Oct;25(10):567-79.

Gold G, Giannakopoulos P, Herrmann FR, Bouras C, Kövari E. Identification of Alzheimer and vascular lesion thresholds for mixed dementia. Brain. 2007; 130 (Pt 11): 2830-6.

González-Alonso J, Dalsgaard MK, Osada T, Volianitis S, Dawson EA, Yoshiga CC, Secher NH. Brain and central haemodynamics and oxygenation during maximal exercise in humans. J Physiol. 2004 May 15;557:331-42.

Gruhn N, Larsen FS, Boesgaard S, Knudsen GM, Mortensen SA, Thomsen G, Aldershvile J. Cerebral blood flow in patients with chronic heart failure before and after heart transplantation. Stroke. 2001; 32(11):2530-3.

Guiraud T, Juneau M, Nigam A, Gayda M, Meyer P, Mekary S, Paillard F, Bosquet L. Optimization of high intensity interval exercise in coronary heart disease. Eur J Appl Physiol. 2010; 108(4):733-40.

Guiraud T, Nigam A, Juneau M, Meyer P, Gayda M, Bosquet L. Acute responses to high-intensity intermittent exercise in CHD patients. Med Sci Sports Exer 2010 b; in press.

The Heart Protection Study: high-risk patients benefit from statins, regardless of LDL-C level. Gurm HS, Hoogwerf B. Cleve Clin J Med. 2003 Nov;70(11):991-7.

Irie F, Fitzpatrick AL, Lopez OL, Kuller LH, Peila R, Newman AB, Launer LJ.Enhanced risk for Alzheimer disease in persons with type 2 diabetes and APOE epsilon4: the Cardiovascular Health Study Cognition Study. Arch Neurol. 2008; 65(1):89-93.

Isomaa B, Almgren P, Tuomi T, Forsen B, Lahti K, Nissen M, et al. Cardiovascular morbidity and mortality associated with the metabolic syndrome. Diabetes Care 2001; 24:683-9.

Clinical trials in mild cognitive impairment: lessons for the future. Jelic V, Kivipelto M, Winblad B. J Neurol Neurosurg Psychiatry. 2006 Apr;77(4):429-38. Epub 2005 Nov 23.

Kivipelto M, Helkala EL, Laakso MP, Hänninen T, Hallikainen M, Alhainen K, Iivonen S, Mannermaa A, Tuomilehto J, Nissinen A, Soininen H. Apolipoprotein E epsilon4 allele, elevated midlife total cholesterol level, and high midlife systolic blood pressure are independent risk factors for late-life Alzheimer disease. Ann Intern Med. 2002 6;137(3):149-55.

Koike A, Itoh H, Oohara R, Hoshimoto M, Tajima A, Aizawa T, Fu LT. Cerebral oxygenation during exercise in cardiac patients. Chest. 2004;125(1):182-90.

Koike A, Hoshimoto M, Nagayama O, Tajima A, Kubozono T, Oikawa K, Uejima T, Momose T, Aizawa T, Fu LT, Itoh H. Cerebral oxygenation during exercise and exercise recovery in patients with idiopathic dilated cardiomyopathy. Am J Cardiol. 2004 15; 94(6):821-4.

Koike A, Hoshimoto M, Tajima A, Nagayama O, Yamaguchi K, Goda A, Yamashita T, Sagara K, Itoh H, Aizawa T. Critical level of cerebral oxygenation during exercise in patients with left ventricular dysfunction. Circ J. 2006; 70(11):1457-61.

Koike A, Nagayama O, Hoshimoto-Iwamoto M, Suzuki T, Tajima A, Uejima T, Aizawa T. Clinical significance of cerebral oxygenation during exercise in patients with coronary artery disease. Circ J. 2008 Nov;72(11):1852-8.

Komulainen P, Lakka TA, Kivipelto M, Hassinen M, Helkala EL, Haapala I, Nissinen A, Rauramaa R. Metabolic syndrome and cognitive function: a population-based follow-up study in elderly women. Dement Geriatr Cogn Disord. 2007; 23(1):29-34.

Kragelj R, Jarm T, Erjavec T, Presern-Strukelj M, Miklavcic D. Parameters of postocclusive reactive hyperemia measured by near infrared spectroscopy in patients with peripheral vascular disease and in healthy volunteers. Ann Biomed Eng. 2001 Apr;29(4):311-20.

Kronenberg G, Bick-Sander A, Bunk E, Wolf C, Ehninger D, Kempermann G. Physical exercise prevents age-related decline in precursor cell activity in the mouse dentate gyrus. Neurobiol Aging. 2006 ; 27(10):1505-13.

Lakka HM, Laaksonen DE, Lakka TA, Niskanen LK, Kumpusalo E, Tuomilehto J, et al. The metabolic syndrome and total and cardiovascular disease mortality in middleaged men. JAMA 2002; 288:2709-16.

Larson EB, Wang L, Bowen JD, McCormick WC, Teri L, Crane P, Kukull W. Exercise is associated with reduced risk for incident dementia among persons 65 years of age and older. Ann Intern Med. 2006 Jan 17;144(2):73-81.

Laursen PB, Jenkins DG. The scientific basis for high-intensity interval training: optimising training programmes and maximising performance in highly trained endurance athletes. Sports Med. 2002;32(1):53-73.

Lautenschlager NT, Cox KL, Flicker L, Foster JK, van Bockxmeer FM, Xiao J, Greenop KR, Almeida OP. Effect of physical activity on cognitive function in older adults at risk for Alzheimer disease: a randomized trial. JAMA. 2008 3;300(9):1027-37.

Lee CW, Lee JH, Kim JJ, Park SW, Hong MK, Kim ST, Lim TH, Park SJ. Cerebral metabolic abnormalities in congestive heart failure detected by proton magnetic resonance spectroscopy. J Am Coll Cardiol. 1999; 33(5):1196-202.

Lee CW, Lee JH, Lim TH, Yang HS, Hong MK, Song JK, Park SW, Park SJ, Kim JJ. Prognostic significance of cerebral metabolic abnormalities in patients with congestive heart failure. Circulation. 2001; 12;103(23): 2784-7.

Lezak, MD, Howieson, D.B., & Loring, D.W. (2004). Neuropsychological Assessment (4th ed.). New York: Oxford University Press

Li G, Rhew IC, Shofer JB, Kukull WA, Breitner JC, Peskind E, Bowen JD, McCormick W, Teri L, Crane PK, Larson EB. Age-varying association between blood pressure and risk of dementia in those aged 65 and older: a community-based prospective cohort study. J Am Geriatr Soc. 2007 Aug;55(8):1161-7.

Lithell H, Hansson L, Skoog I, Elmfeldt D, Hofman A, Olofsson B, Trenkwalder P, Zanchetti A; SCOPE Study Group. The Study on Cognition and Prognosis in the Elderly (SCOPE): principal results of a randomized double-blind intervention trial. J Hypertens. 2003 May;21(5):875-86.

Liu CY, Zhou HD, Xu ZQ, Zhang WW, Li XY, Zhao J. Metabolic syndrome and cognitive impairment amongst elderly people in Chinese population: a cross-sectional study. Eur J Neurol. 2009 Sep;16(9):1022-7.

Luck T, Luppa M, Briel S, Riedel-Heller SG. Incidence of Mild Cognitive Impairment: A Systematic Review. Dement Geriatr Cogn Disord. 2010; 29(2):164-175.

Molinari F, Liboni W, Grippi G, Negri E. Relationship between oxygen supply and cerebral blood flow assessed by transcranial Doppler and near-infrared spectroscopy in healthy subjects during breath-holding. J Neuroeng Rehabil. 2006 19;3:16.

Nagayama O, Koike A, Hoshimoto M, Yamaguchi K, Tajima A, Goda A, Uejima T, Itoh H, Aizawa T. Influence of cerebrovascular arteriosclerosis on cerebral oxygenation during exercise. Circ J. 2007 May;71(5):782-7.

Nigam A, Bourassa M, Fortier A, Guertin MC, Tardif JC. The metabolic syndrome and its components and the long-term risk of death in patients with coronary heart disease. Am Heart J 2006; 151: 514-21.

Ott A, Stolk RP, Hofman A, van Harskamp F, Grobbee DE, Breteler MM. Association of diabetes mellitus and dementia: the Rotterdam Study. Diabetologia. 1996; 39(11):1392-7

Oveisgharan S, Hachinski V. Hypertension, executive dysfunction, and progression to dementia: the canadian study of health and aging. Arch Neurol. 2010; 67(2):187-92

Paul RH, Gunstad J, Poppas A, Tate DF, Foreman D, Brickman AM, Jefferson AL, Hoth K, Cohen RA. Neuroimaging and cardiac correlates of cognitive function among patients with cardiac disease. Cerebrovasc Dis. 2005; 20(2):129-33.

Peltonen JE, Paterson DH, Shoemaker JK, Delorey DS, Dumanoir GR, Petrella RJ, Kowalchuk JM. Cerebral and muscle deoxygenation, hypoxic ventilatory chemosensitivity and cerebrovascular responsiveness during incremental exercise. Respir Physiol Neurobiol. 2009;169(1):24-35.

Perrey S. Non-invasive NIR spectroscopy of human brain function during exercise. Methods. 2008 ;45(4):289-99.

Petersen RC, Thomas RG, Grundman M, Bennett D, Doody R, Ferris S, Galasko D, Jin S, Kaye J, Levey A, Pfeiffer E, Sano M, van Dyck CH, Thal LJ; Alzheimer's Disease Cooperative Study Group. Vitamin E and donepezil for the treatment of mild cognitive impairment. N Engl J Med. 2005 Jun 9;352(23):2379-88.

Podewils LJ, Guallar E, Kuller LH, Fried LP, Lopez OL, Carlson M, Lyketsos CG. Physical activity, APOE genotype, and dementia risk: findings from the Cardiovascular Health Cognition Study. Am J Epidemiol. 2005 1;161(7):639-51.

Purandare N. Preventing dementia: role of vascular risk factors and cerebral emboli. Br Med Bull. 2009; 91:49-59.

Richard R, Lonsdorfer-Wolf E, Charloux A, Doutreleau S, Buchheit M, Oswald-Mammosser M, Lampert E, Mettauer B, Geny B, Lonsdorfer J. Non-invasive cardiac output evaluation during a maximal progressive exercise test, using a new impedance cardiograph device. Eur J Appl Physiol. 2001; 85(3-4):202-7.

Roberts RO, Knopman DS, Geda YE, Cha RH, Roger VL, Petersen RC. Coronary heart disease is associated with non-amnestic mild cognitive impairment. Neurobiol Aging. 2008 Dec 15. [Epub ahead of print].

Rognmo Ø, Hetland E, Helgerud J, Hoff J, Slørdahl SA. High intensity aerobic interval exercise is superior to moderate intensity exercise for increasing aerobic capacity in patients with coronary artery disease. Eur J Cardiovasc Prev Rehabil. 2004 ;11(3):216-22.

Rosengren A, Hawken S, Ounpuu S, Sliwa K, Zubaid M, Almahmeed WA, et al. Association of psychosocial risk factors with risk of acute myocardial infarction in 11 119 cases and 13 648 controls from 52 countries (the INTERHEART study): case-control study. Lancet 2004;364:953-62.

Rovio S, Kåreholt I, Helkala EL, Viitanen M, Winblad B, Tuomilehto J, Soininen H, Nissinen A, Kivipelto M. Leisure-time physical activity at midlife and the risk of dementia and Alzheimer's disease. Lancet Neurol. 2005; 4(11):705-11.

Rupp T, Perrey S. Prefrontal cortex oxygenation and neuromuscular responses to exhaustive exercise. Eur J Appl Physiol. 2008 Jan;102(2):153-63.

Safonova LP, Michalos A, Wolf U, Wolf M, Hueber DM, Choi JH, Gupta R, Polzonetti C, Mantulin WW, Gratton E. Age-correlated changes in cerebral hemodynamics assessed by near-infrared spectroscopy. Arch Gerontol Geriatr. 2004; 39(3):207-25.

Singh-Manoux A, Britton AR, Marmot M. Vascular disease and cognitive function: evidence from the Whitehall II Study. J Am Geriatr Soc. 2003; 51(10):1445-50.

Skoog I, Lernfelt B, Landahl S, Palmertz B, Andreasson LA, Nilsson L, Persson G, Odén A, Svanborg A. 15-year longitudinal study of blood pressure and dementia. Lancet. 1996 Apr 27;347(9009):1141-5.

Soneira CF, Scott TM. Severe cardiovascular disease and Alzheimer's disease: senile plaque formation in cortical areas. Clin Anat. 1996; 9(2):118-27.

Sparks DL, Hunsaker JC 3rd, Scheff SW, Kryscio RJ, Henson JL, Markesbery WR. Cortical senile plaques in coronary artery disease, aging and Alzheimer's disease. Neurobiol Aging. 1990;11(6):601-7.

Statistics Canada: http://www.statcan.gc.ca/pub/84-215-x/2008000/tbl-eng.htm: leading causes of death in Canada.

Subudhi AW, Dimmen AC, Roach RC. Effects of acute hypoxia on cerebral and muscle oxygenation during incremental exercise. J Appl Physiol. 2007; 103(1):177-83.

Subudhi AW, Lorenz MC, Fulco CS, Roach RC. Cerebrovascular responses to incremental exercise during hypobaric

hypoxia: effect of oxygenation on maximal performance. Am J Physiol Heart Circ Physiol. 2008; 294(1):H164-71.

Swain RA, Harris AB, Wiener EC, Dutka MV, Morris HD, Theien BE, Konda S, Engberg K, Lauterbur PC, Greenough WT. Prolonged exercise induces angiogenesis and increases cerebral blood volume in primary motor cortex of the rat. Neuroscience. 2003;117(4):1037-46.

Tanne D, Freimark D, Poreh A, Merzeliak O, Bruck B, Schwammenthal Y, Schwammenthal E, Motro M, Adler Y. Cognitive functions in severe congestive heart failure before and after an exercise training program. Int J Cardiol. 2005 Aug 18;103(2):145-9.

Thal LJ, Ferris SH, Kirby L, Block GA, Lines CR, Yuen E, Assaid C, Nessly ML, Norman BA, Baranak CC, Reines SA; Rofecoxib Protocol 078 study group. A randomized, double-blind, study of rofecoxib in patients with mild cognitive impairment. Neuropsychopharmacology. 2005 Jun;30(6):1204-15.

Tjønna AE, Lee SJ, Rognmo Ø, Stølen TO, Bye A, Haram PM, Loennechen JP, Al-Share QY, Skogvoll E, Slørdahl SA, Kemi OJ, Najjar SM, Wisløff U. Aerobic interval training versus continuous moderate exercise as a treatment for the metabolic syndrome: a pilot study. Circulation. 2008 Jul 22;118(4):346-54.

Trojano L, Antonelli Incalzi R, Acanfora D, Picone C, Mecocci P, Rengo F; Congestive Heart Failure Italian Study Investigators. Cognitive impairment: a key feature of congestive heart failure in the elderly. J Neurol. 2003; 250(12):1456-63.

Turcotte M, Schellenberg G 2006: Statistique Canada: Un portrait des aînés au Canada. http://www.statcan.gc.ca/pub/89-519-x/89-519-x2006001-fra.pdf

Uda M, Ishido M, Kami K, Masuhara M. Effects of chronic treadmill running on neurogenesis in the dentate gyrus of the hippocampus of adult rat. Brain Res. 2006 9;1104(1):64-72.

Van Beekvelt MC, Colier WN, Wevers RA, Van Engelen BG. Performance of near-infrared spectroscopy in measuring local O(2) consumption and blood flow in skeletal muscle. J Appl Physiol. 2001; 90(2):511-9.

van Gelder BM, Tijhuis MA, Kalmijn S, Giampaoli S, Nissinen A, Kromhout D. Physical activity in relation to cognitive decline in elderly men: the FINE Study. Neurology. 2004 28; 63(12):2316-21.

Van Der Sluijs MC, Colier WNJM, Houston RJF, Oeseburg B. A new and highly sensitive continuous wave near infrared spectrophotometer with multiple detectors. Benaron D.A., Chance B. & Ferrari M. (1998); 3194: (SPIE. Proc), 63–72. Photon Propagation in Tissues III.

Vanhanen M, Koivisto K, Moilanen L, Helkala EL, Hänninen T, Soininen H, Kervinen K, Kesäniemi YA, Laakso M, Kuusisto J. Association of metabolic syndrome with Alzheimer disease: a population-based study. Neurology. 2006 12; 67(5):843-7.

Warburton DE, McKenzie DC, Haykowsky MJ, Taylor A, Shoemaker P, Ignaszewski AP, Chan SY. Effectiveness of high-intensity interval training for the rehabilitation of patients with coronary artery disease. Am J Cardiol. 2005 1;95(9):1080-4.

Weuve J, Kang JH, Manson JE, Breteler MM, Ware JH, Grodstein F. Physical activity, including walking, and cognitive function in older women. JAMA. 2004 22; 292(12):1454-61.

Wisløff U, Støylen A, Loennechen JP, Bruvold M, Rognmo Ø, Haram PM, Tjønna AE, Helgerud J, Slørdahl SA, Lee SJ, Videm V, Bye A, Smith GL, Najjar SM, Ellingsen Ø, Skjaerpe T. Superior cardiovascular effect of aerobic interval training versus moderate continuous training in heart failure patients: a randomized study. Circulation. 2007 19; 115(24):3086-94.

Working Group on Cardiac Rehabilitation & Exercice Physiology and Working Group on Heart Failure of the European Society of Cardiology. Recommendations for exercise training in chronic heart failure patients. Eur Heart J. 2001; 22(2):125-35. Review.

Woo MA, Macey PM, Fonarow GC, Hamilton MA, Harper RM. Regional brain gray matter loss in heart failure. J Appl Physiol. 2003; 95(2):677-84.

Yaffe K, Haan M, Blackwell T, Cherkasova E, Whitmer RA, West N. Metabolic syndrome and cognitive decline in elderly

Latinos: findings from the Sacramento Area Latino Study of Aging study. J Am Geriatr Soc. 2007; 55(5):758-62.

Yaffe K, Kanaya A, Lindquist K, Simonsick EM, Harris T, Shorr RI, Tylavsky FA, Newman AB. The metabolic syndrome, inflammation, and risk of cognitive decline. JAMA. 2004 10; 292(18):2237-42.

Yaffe K, Weston AL, Blackwell T, Krueger KA. The metabolic syndrome and development of cognitive impairment among older women. Arch Neurol. 2009; 66(3):324-8.

Yaffe K. Metabolic syndrome and cognitive disorders: is the sum greater than its parts? Alzheimer Dis Assoc Disord. 2007; 21(2):167-71.

Yesavage. JA, Brink TL, Rose TL, Lum O, Huang V, Adey M, Leirer VO. Development and validation of a geriatric depression screening scale: a preliminary report. J Psychiatr Res. 1982-1983;17(1):37-49.

Zuccalà G, Pedone C, Cesari M, Onder G, Pahor M, Marzetti E, Lo Monaco MR, Cocchi A, Carbonin P, Bernabei R. The effects of cognitive impairment on mortality among hospitalized patients with heart failure. Am J Med. 2003 1;115(2):97-103.

ANNEXE 1 : UTILISATION DE LA BIOBANQUE DE L'ICM AUX FINS DU PROJET

☐ **SANS OBJET**

1. **S'agit-il d'une recherche dans le cadre d'une collaboration avec un chercheur externe ?**

   ☐ Oui ☐ Non

   Si oui, indiquer le nom et l'affiliation du (des) chercheur(s) externe(s) :

   Sélectionnez titreNom, Prénom

   Centre d'affiliation :

   Sélectionnez titreNom, Prénom

   Centre d'affiliation :

2. **Informations sur les donnés / matériel biologiques :**

Nombre de **cas** visés :	
Nombre de **témoins** visés :	
Durée de l'utilisation :	
Type de données désirées (Joindre la liste des variables demandées)	☐ Dénominalisées ☐ Anonymisées
Type de matériel biologique désiré et quantité du matériel biologique	☐ ADN Quantité :<br>☐ Plasma Quantité :<br>☐ Cellules rouges Quantité :<br>☐ Autre :<br>Quantité :
Données transférées à l'extérieur du Canada ?<br>Si oui, quel pays ?<br>Commentaires :	☐ Oui ☐ Non
Matériel biologique transféré à l'extérieur du Canada ?<br>Si oui, quel pays ?<br>Commentaire :	☐ Oui ☐ Non ☐ N/A
Sera-t-il nécessaire de recontacter les participants de la cohorte de l'ICM ?	☐ Oui ☐ Non

ESPACE RÉSERVÉ AU COMITÉ DE GESTION

APPROBATION			
Projet révisé par le Comité de gestion à la réunion du :	Jour	Mois	Année
Personnes présentes lors de la réunion :			
☐**Accepté, le projet peut être soumis au CÉRDNT**<br>☐**Refusé, raison :**			
Nom et signature du Directeur du Comité de gestion :	Jour	Mois	Année

FORMULAIRE DE CONSENTEMENT

PROJET DE RECHERCHE : ICM #10-1240

Exercice, débit cardiaque, oxygénation cérébrale, et performance cognitive chez les patients présentant un syndrome métabolique, une maladie coronarienne, ou une insuffisance cardiaque.

COGNEX

Investigateur principal et collaborateurs

Anil Nigam, M.D, MS.C, Mathieu Gayda, Ph.D, Vincent Gremeaux, M.D, MS.C, Martin Juneau, M.D, Frédéric Lesage, Ph.D, Eric Thorin, Ph.D, Jean Lambert, Ph.D, Louis Bherer, Ph.D, Sarah Fraser, Ph.D, Olivier Dupuy, Ph.D, Gabriel Lapierre, B.Sc, Véronique Labelle, M.Ps.

Commanditaire : Les fonds de recherche des Docteurs Martin Juneau et Anil Nigam

INFORMATION

DESCRIPTION GÉNÉRALE

Nous vous invitons à participer à une étude clinique subventionnée par les fonds de recherche des Docteurs Juneau et Nigam, parce que vous présentez soit un syndrome métabolique, une maladie coronarienne ou une insuffisance cardiaque. Vous pourriez également être un participant du groupe de contrôle, c'est à dire sans aucune des conditions ci-haut mentionnées.

Ce formulaire de consentement décrit les procédures que vous devrez suivre si vous acceptez de participer à cette étude.

Avant de signer ce formulaire de consentement, veuillez prendre tout le temps nécessaire pour lire (ou vous faire lire) et comprendre l'information présentée ci-dessous. Vous pouvez consulter vos proches avant de prendre votre décision. Veuillez poser à votre médecin ou à l'équipe de recherche toutes les questions que vous avez sur la présente étude et sur vos droits. Ils devraient être en mesure de répondre à toutes vos questions.

La participation simultanée à plusieurs études peut être dangereuse pour vous. Si vous participez déjà à une étude clinique, veuillez en aviser votre médecin.

Le tableau présenté à la page suivante nomme et définit les trois conditions énumérées et celles du groupe de contrôle. Le groupe auquel vous appartenez est coché.

Tableau : Les trois conditions énumérées et celles du groupe de contrôle.

**Condition du participant (cocher)**	**Condition**	**Définition**
	syndrome métabolique	Le syndrome métabolique est défini par la présence simultanée de plusieurs des éléments suivants: tour de taille augmenté ; perturbations du bilan des lipides sanguins (cholestérol et triglycérides); augmentation de la pression artérielle ; taux de sucre dans le sang à jeun augmenté.
	maladie coronarienne	La maladie coronarienne résulte de l'accumulation de facteurs de risques cardiovasculaires, tels que l'hypertension artérielle, le surpoids, la sédentarité, les troubles lipidiques et le diabète. Elle expose à des complications telles que l'infarctus du myocarde.
	insuffisance cardiaque	L'insuffisance cardiaque est due à une incapacité du cœur à assurer le débit sanguin nécessaire au fonctionnement des différents organes (muscles, cerveau, viscères…). Elle constitue une complication fréquente chez les patients souffrants d'une atteinte des artères coronaires, en particulier ceux qui ont présenté un infarctus.
	groupe de contrôle	Pour faire partie du groupe de contrôle vous ne devez pas présenter au moment de l'étude :<br>▪ un événement cardiaque instable non contrôlé (angine de poitrine instable, infarctus en cours);<br>▪ avoir d'antécédent de pathologie cardiaque;<br>▪ être insuffisant cardiaque;<br>▪ présenter un syndrome métabolique, défini par la présence simultanée de plusieurs des éléments suivants: tour de augmenté ; perturbations du bilan des lipides sanguins (cholestérol et triglycérides); augmentation de la pression artérielle ; taux de sucre dans le sang à jeun augmenté.

But de l'étude :

Toutes ces pathologies exposent à une baisse des performances cognitives (mémoire, raisonnement abstrait, calcul...) plus marquées que celle liée au vieillissement normal chez le sujet sain.

Initiales du patient: ________________

Le but de ce travail est donc d'étudier les relations entre la fonction cognitive, l'oxygénation cérébrale, le débit cardiaque, et la tolérance à l'effort, chez des patients présentant un syndrome métabolique, une maladie coronarienne, ou une insuffisance cardiaque.

Les mécanismes supposés sont la baisse du débit cardiaque et du flot sanguin au cerveau. Les traitements médicamenteux sont peu efficaces pour lutter contre la diminution des performances cognitives. Par contre, les effets de l'activité physique dans ce domaine sont encourageants. Des travaux montrent que l'activité physique régulière induit un effet protecteur sur le développement ou l'évolution de troubles cognitifs.

Les mécanismes induisant ces effets restent mal compris. Une des hypothèses est celle d'une amélioration du fonctionnement des artères du cerveau, et donc de la perfusion cérébrale, en réaction à la pratique d'une activité physique régulière.

L'oxygénation cérébrale peut être étudiée avec un appareil totalement inoffensif et indolore utilisant l'infra-rouge (technologie NIRS). Des études ont ainsi montré que lors de l'effort, l'oxygénation cérébrale évolue différemment entre les sujets sains et des patients souffrant de pathologies cardio-respiratoires (diminution du flot sanguin au cerveau dès le début de l'effort). Cependant, les relations entre l'oxygénation cérébrale à l'effort, les performances cognitives et physiques n'ont jamais été étudiées à ce jour.

Les tests seront effectués :

1. Lors d'un exercice maximal. (Volet 1)

2. Pendant et après une période d'entraînement de 12 semaines, selon 2 modalités possibles: entrainement continu d'intensité modérée ou entraînement par intervalles à haute intensité (Volet 2).

Cette étude permettra de mieux comprendre les mécanismes impliqués :

- dans l'apparition des troubles cognitifs chez les patients présentant un syndrome métabolique, une maladie coronarienne, ou une insuffisance cardiaque ;

- dans l'amélioration de ces troubles par l'exercice physique régulier. Ces résultats devraient permettre de mieux individualiser les programmes de réentraînement afin d'optimiser l'amélioration de la qualité de vie de ces patients.

Un total de 140 participants (50 présentant un syndrome métabolique, 25 avec une maladie cardiaque, 20 avec de l'insuffisance cardiaque et 45 du groupe de contrôle) prendront part au volet 1 de cette étude au centre EPIC.

Un total de 160 participants (40 présentant un syndrome métabolique, 40 avec une maladie cardiaque, 40 avec de l'insuffisance cardiaque et 40 du groupe de contrôle) prendront part au volet 2 de cette étude au centre EPIC.

DÉROULEMENT DE L'ÉTUDE

L'étude comporte deux volets. Il y aura quatre (4) visites pour le volet 1. Ces quatre (4) visites seront suivies d'un période d'entraînement de douze (12) semaines pour les participants candidats au volet 2.

VOLET 1 :

La première visite servira à vous présenter le formulaire de consentement et à vous renseigner sur l'étude. Si vous êtes d'accord pour participer à l'étude, vous verrez un médecin qui recueillera votre historique médical, nous mesurerons aussi votre taille, votre poids et votre composition corporelle (masse musculaire et masse grasse du corps) et nous procéderons également à une prise de sang pour analyse sanguine (cholestérol, sucre dans le sang).

Au cours de cette visite, vous aurez également une mesure de vos capacités de dilatation des artères au niveau du bras (test d'hyperhémie brachiale) et du cerveau (test de breath-holding ou rétention de votre souffle). La durée de cette visite sera d'environ 1 heure.

Pour le test d'hyperhémie brachiale, vous serez installé dans un fauteuil confortable et devrez placer vos deux bras sur les accoudoirs du fauteuil. Une compression sera appliquée sur un bras (bras droit) par un brassard de tension artérielle pour faire un examen d'hyperhémie réactive brachiale.

Une première pression artérielle de repos sera notée. Par la suite, la sonde de la spectroscopie proche de l'infra rouge (NIRS) sera installée avec un adhésif sur votre bras (partie intérieure) entre le poignet et le coude. Une mesure de 3 minutes sera réalisée pour avoir les valeurs de repos.

L'appareil à pression sera alors gonflé à une faible pression (50 mmHg) pendant 2 périodes de 1 minute, séparées d'une minute de repos, pour bloquer la circulation veineuse de votre bras. Après 2 minutes de repos, le brassard sera à nouveau gonflé, plus haut que votre pression artérielle (60 mmHg de plus) afin de bloquer la circulation artérielle de votre bras pendant 5 minutes. Quand le brassard est dégonflé, un afflux de sang se produit. Après le dégonflage, la sonde de NIRS continuera à enregistrer l'oxygénation pendant 5 minutes.

Pour le test de breath-holding, vous serez installé dans le même fauteuil, avec la sonde de NIRS installée avec un adhésif sur la partie gauche de votre front. Une mesure de 3 minutes sera réalisée pour avoir les valeurs de repos.

Puis il vous sera demandé de retenir votre souffle à la fin d'une expiration normale, pour une durée minimale de 15 secondes, voire plus si vous sous sentez confortable. Vous répéterez cette manœuvre à la fin d'une inspiration forcée quelques minutes plus tard, puis la sonde de NIRS continuera à enregistrer l'oxygénation pendant 5 minutes. Après 5 minutes de repos, ces deux mesures (en expiration et en inspiration) seront répétées une fois.

La deuxième visite est pour faire le bilan cognitif. Ce bilan a été élaboré en collaboration avec des chercheurs spécialisés dans la mémoire et le fonctionnement cérébral. Il se compose de tests neuropsychologiques auxquels il vous sera demandé de répondre le mieux possible. Ils seront réalisés soit avec un papier et un crayon, soit sur ordinateur. Certains seront chronométrés. Ce bilan sera réalisé avec une personne maîtrisant l'administration ces tests, dans une salle calme, et durera environ 1 heure.

La troisième visite est une épreuve d'effort sur vélo avec analyse des gaz expiratoires, et enregistrement en continu de l'électrocardiogramme, du débit cardiaque, et de l'oxygénation cérébrale. La mesure du débit cardiaque est effectuée à l'aide d'un appareil totalement indolore, appelé bio-impédance cardiaque, qui nécessite la pose de 6 électrodes supplémentaires sur la peau du thorax.

La mesure de l'oxygénation cérébrale sera faite par la sonde NIRS placée sur la partie gauche de votre front. Cette épreuve d'effort sera réalisée sous la supervision d'un kinésiologue ou d'une infirmière, et d'un médecin, et durera environ 1 heure.

La quatrième visite comportera la réalisation d'un test cognitif court pendant l'effort. Vous serez installé sur le même vélo que lors de la 3ème visite. Après un échauffement de 5 minutes, vous effectuerez deux périodes de pédalages, une d'intensité modérée, et une plus intense, mais restant en dessous de votre maximum. Il vous sera demandé de refaire un des tests cognitifs de la 2ème visite au cours de chacune de ces 2 périodes de pédalage. Ce test permettra d'évaluer l'impact de l'intensité de l'effort sur la performance cognitive. Cette visite durera environ 30 minutes.

Si vous encourez des dépenses pour participer à ce projet de recherche (ex. Déplacements, repas, etc.), veuillez discuter avec l'infirmière de recherche de la possibilité d'en obtenir le remboursement et de la procédure à suivre.

VOLET 2 :

Le volet 2 sera proposé aux patients débutant l'entraînement au centre EPIC, après une période de familiarisation de 2 semaines. Il vous sera alors proposé de participer à un programme d'entraînement d'une durée de 12 semaines à raison de 3 à 5 séances par semaines.

Deux modalités d'entraînement habituellement pratiquées au centre EPIC seront proposées. La modalité sera choisie aléatoirement, c'est-à-dire au hasard, en fonction de votre ordre d'inclusion dans l'étude :

- entraînement continu d'intensité modérée
- entraînement par intervalles à haute intensité.

Ces 2 modalités ont montré leur efficacité pour améliorer les capacités physiques et le contrôle des facteurs de risques chez les patients présentant des facteurs de risques cardiovasculaires. La modalité par intervalles semble perçue comme plus confortable par les patients, même si les sociétés savantes de cardiologie recommandent encore à l'heure actuelle un entrainement continu. Tous les entrainements seront effectués sur un vélo (bicyclette stationnaire) sous la supervision d'un kinésiologue expérimenté et d'un médecin.

À la fin des douze (12) semaines d'entrainement, les quatre visites du volet 1 seront répétées.

En complément, une mesure ambulatoire de votre pression artérielle (MAPA) va être réalisée durant 24h après une session d'exercice, ceci à 2 reprises : 1 fois en début d'entraînement, puis 1 fois à la fin des 12 semaines d'entraînement, afin de mesurer le bénéfice de l'entraînement sur votre pression artérielle. La MAPA est un enregistrement de votre pression artérielle pendant 24h à l'aide d'un appareil portable que vous emmènerez à votre domicile. La mesure de votre pression artérielle sera réalisée automatiquement toutes les 20 minutes. Il vous sera demandé de retranscrire sur une fiche, vos activités au cours de ces 24h et de vous présenter le lendemain de la pose pour ramener l'appareil.

RISQUES ET INCONVÉNIENTS

Risques liés au test d'hyperhémie brachiale et breath-holding avec spectroscopie proche de l'infra rouge (NIRS).

Il n'y a pas de risques réels liés au test d'hyperhémie brachiale avec spectroscopie proche de l'infra rouge (NIRS). Pour ce qui est de la compression artérielle par le brassard, il occasionne un inconfort semblable à la prise de la tension artérielle, mais pendant quelques minutes. Dans certains cas, le bras peut devenir engourdi ou douloureux. Lors du relâchement de la compression artérielle, vous sentirez des picotements dans votre bras pour une durée d'environ 2-3 minutes. En

ce qui concerne le breath-holding, vous pourrez ressentir un inconfort lié à la courte apnée. En cas d'impossibilité de tenir 15 secondes, il vous sera demandé un 2e essai.

Quelques patients peuvent éprouver une sensation de faiblesse et d'évanouissement pendant la procédure. Cette réaction est transitoire et est probablement causée par l'anxiété. Si cela survient, vous serez rapidement placé en position couchée pour favoriser votre récupération. La durée totale du test sera d'environ 40 minutes.

Risques lies à la prise de sang

La prise de sang veineuse pour analyse du cholestérol est une pratique médicale courante, mais peut être temporairement désagréable. Vous pourriez voir une décoloration ou une ecchymose au niveau du site de la piqûre. Très rarement, une infection pourrait apparaître au niveau de la zone de la piqûre.

Risques liés à l'épreuve d'effort sur vélo
Le principal risque est la survenue d'un événement cardiaque grave comme un infarctus du myocarde. Ce risque est faible, approximativement 1/10,000, et sera encore diminué par votre entrevue préalable avec le médecin au cours de la visite 1, qui ne vous inclura pas s'il pense qu'il existe un risque. Par ailleurs, certains patients peuvent ressentir une sensation de faiblesse passagère ou d'évanouissement à l'arrêt de l'effort. Cette réaction est transitoire et le plus souvent bénigne. Un médecin sera présent pour vous surveiller pendant toute la durée du test, et vous prendre en charge si besoin.

Risques liés à l'entraînement
L'entraînement sur vélo à haute intensité peut très rarement causer de complications cardio-vasculaires majeures (telles qu'une perte de conscience ou un infarctus du myocarde). Le risque est très faible, il est estimé à 1/60,000 par heure d'entraînement.

Risques liés à la mesure ambulatoire de pression artérielle (MAPA) des 24h
Cet examen peut être parfois gênant mais est indolore. Le brassard peut serrer fort et faire une rougeur au niveau du bras mais sans conséquence.

AVANTAGES

VOLET 1 :
Vous ne retirerez aucun bénéfice direct en participant à cette étude. Toutefois, votre participation à ce projet de recherche contribuera à l'avancement des connaissances sur la relation entre les capacités physiques, les facteurs de risques cardio-vasculaires, et le fonctionnement cérébral.

VOLET 2 :
Vous bénéficierez d'un programme d'entraînement qui, quelle que soit la modalité, a fait les preuves de son efficacité dans la prévention des maladies cardio-vasculaires et le contrôle des facteurs de risques cardio-vasculaires. Ce programme participe également à l'amélioration de votre qualité de vie par de meilleures capacités physiques et un effet positif direct sur l'humeur. De plus, votre participation à ce projet de recherche permettra de déterminer les modalités d'entraînement optimal et de les généraliser dans la pratique quotidienne pour les autres patients présentant un syndrome métabolique.

PARTICIPATION VOLONTAIRE

Vous êtes libre de participer à cette étude ou de vous en retirer en tout temps sur simple avis verbal sans avoir à préciser les motifs de votre décision. Si vous décidez de ne pas y participer ou de vous en retirer, vous recevrez les soins médicaux usuels pour le traitement de votre condition.

Quelle que soit votre décision, celle-ci n'influencera en rien la qualité des soins que vous êtes en droit de recevoir.

Vous serez informé de toute nouvelle découverte importante faite au cours de l'étude et susceptible d'influencer votre décision de maintenir ou non votre participation à l'étude.

Il se pourrait également qu'en apprenant de nouveaux faits, votre médecin décide qu'il vaut mieux pour vous que vous soyez retiré de l'étude. Le cas échéant, il vous donnera des explications et prendra des mesures pour la poursuite de vos traitements.

CONFIDENTIALITÉ

Durant votre participation à cette étude, l'équipe de recherche consultera votre dossier médical et recueillera des données personnelles et de santé (histoire médicale, examen physique, résultats de laboratoire) afin de réaliser ce projet de recherche.

Tous les renseignements obtenus seront strictement confidentiels (à moins d'une autorisation de votre part à les communiquer à d'autres personnes ou d'une exception de la loi nous autorisant à les communiquer).

L'équipe de recherche utilisera vos données et les analysera avec les données des autres participants pour réaliser ce projet de recherche. Pour protéger votre identité, vos données personnelles ne seront identifiées que par un code qui vous sera assigné en remplacement de votre nom. Les données révélant votre identité sont conservées à l'ICM sous la responsabilité de Dr Gayda. Tous les dossiers de recherche seront conservés sous clé et dans des fichiers sécurisés pendant 25 ans.

Aux fins de s'assurer du bon déroulement du projet, il est possible qu'un délégué du comité d'éthique de la recherche ou un organisme réglementaire tel que Santé Canada consultent les données de recherche et votre dossier médical.

Les résultats de cette étude seront publiés et diffusés mais aucune information permettant de vous identifier ne sera alors dévoilée.

Registre du ministère

Le Ministère de la Santé et des Services sociaux du Québec exige que l'Institut de Cardiologie de Montréal tienne un registre des personnes qui participent à des projets de recherche pour fins de sécurité, de contrôle des risques, d'inspection et de statistiques. Votre nom et votre numéro de dossier médical figureront dans ce registre. Vous pouvez accéder en tout temps aux données qui vous concernent pour en connaître le contenu et le faire rectifier au besoin.

COMPENSATION

Dans l'éventualité où vous seriez victime d'un préjudice causé par le médicament à l'étude ou par toute autre procédure ou technologie requise par le protocole de recherche, le Dr Gayda veillera à ce que vous receviez tous les soins que nécessite votre état de santé.

Si votre participation engendrait d'autres coûts qui ne sont pas présentement assurés par les régimes d'assurance-hospitalisation et d'assurance-maladie du Québec, ceux-ci ne sont pas couverts. Vous devrez donc en débourser les frais. De plus, aucune compensation pour perte de revenus, invalidité ou inconfort n'est prévue.

Toutefois, en signant ce formulaire de consentement, vous ne renoncez à aucun de vos droits. Notamment, vous ne libérez pas l'investigateur de ses responsabilités légales et professionnelles advenant une situation qui vous causerait préjudice.

IDENTIFICATION DES PERSONNES-RESSOURCES

Si vous avez des questions concernant le projet de recherche ou si vous éprouvez un problème que vous croyez relié à votre participation au projet de recherche, vous pouvez communiquer avec le chercheur responsable du projet de recherche aux numéros suivants :

Vous pouvez communiquer en tout temps avec :

Institut de Cardiologie de Montréal, Centre EPIC
Dr Anil Nigam, cardiologue, Chercheur principal : Tél. : (514) 376-3330, poste 3010
Dr Gremeaux : Tél. : (514) 374-1480, poste 272

Madame Julie Lalongé : Tél. : (514) 374-1480, poste 259
Madame Marie Cournoyer : Tél. : (514) 374-1480, poste 252

Pour toute question concernant vos droits en tant que sujet participant à ce projet de recherche ou si vous avez des plaintes ou des commentaires à formuler vous pouvez communiquer avec le commissaire local aux plaintes et à la qualité des services de l'Institut de Cardiologie de Montréal au numéro suivant : (514) 376-3330 poste 3398.

Initiales du patient: ________________

FORMULAIRE DE CONSENTEMENT

PROJET DE RECHERCHE : ICM #10-1240

Exercice, débit cardiaque, oxygénation cérébrale, et performance cognitive chez les patients présentant un syndrome métabolique, une maladie coronarienne, ou une insuffisance cardiaque.

COGNEX

Investigateur principal et collaborateurs

Anil Nigam, M.D, MS.C, Mathieu Gayda, Ph.D, Vincent Gremeaux, M.D, MS.C, Martin Juneau, M.D, Frédéric Lesage, Ph.D, Eric Thorin, Ph.D, Jean Lambert, Ph.D, Louis Bherer, Ph.D, Sarah Fraser, Ph.D, Olivier Dupuy, Ph.D, Gabriel Lapierre, B.Sc, Véronique Labelle, M.Ps.

Commanditaire : Les fonds de recherche des Docteurs Martin Juneau et Anil Nigam

J'ai eu l'occasion de poser toutes les questions voulues au sujet de ce projet et on y a répondu à ma satisfaction.

Je comprends que je demeure libre de me retirer de ce projet en tout temps sans que cela n'affecte en aucune façon les soins dont je pourrais bénéficier à l'avenir.

J'ai lu ou l'on m'a lu ce formulaire de consentement et j'en comprends le contenu.

Je, soussigné(e), accepte de participer au présent projet de recherche.

J'accepte que mon médecin de famille soit informé de ma participation à ce projet : ☐**oui** ☐**non**

Signature du patient	*Nom du patient en lettres moulées*	*Date (a/m/j)*	*Heure*

Signature de l'un des chercheurs	*Nom du chercheur en lettres moulées*	*Date (a/m/j)*	*Heure*

Je certifie que j'ai expliqué les buts du projet à ________________________et il(elle) a signé le consentement en ma présence.

Signature du chercheur ou de son délégué	*Nom du chercheur ou de son délégué en lettres moulées*	*Date (a/m/j)*	*Heure*

Le Comité d'éthique de la recherche et du développement des nouvelles technologies de l'Institut de Cardiologie de Montréal autorise le début du recrutement en date du 5 janvier 2011. La version courante no. 4 du consentement en français datée du 18 décembre 2013 est approuvée.

N.B. : L'original de ce formulaire doit être inséré au dossier du patient, une copie gardée par l'investigateur et une copie remise au patient.
