# Supplementary material for: Cognitive function in patients with stable coronary heart disease: Related cerebrovascular and cardiovascular responses
Source: PLoS One. 2017 Sep 22;12(9):e0183791. doi: 10.1371/journal.pone.0183791 (PMC5609740; doi:10.1371/journal.pone.0183791)
Supplement: S5 File — (PDF) [file pone.0183791.s005.pdf]

INSTITUT DE CARDIOLOGIE DE MONTRÉAL

AFFILIÉ À Université de Montréal

Formulaire de notification d'une déviation au protocole de recherche susceptible de remettre en cause l'éthicité d'un projet de recherche

Date de dépôt du formulaire: **2013-12-03 14:55**

Date d'approbation du projet: **2011-01-06**

Numéro(s) de projet: **2010-154, 1240**

Déposé par: **Lalongé, Julie**

Identifiant nagano: **COGNEX**

Formulaire: **F8 - 1673**

Renseignements généraux

1. **Indiquez, en français, le titre complet du projet de recherche.**

   Oxygénation cérébrale, débit cardiaque, performance cognitive et exercice chez les patients présentant un syndrome métabolique, une maladie coronarienne, ou une insuffisance cardiaque.

2. **Indiquez le nom du chercheur principal.**

   Nigam, Anil

3. **Indiquez, le cas échéant des collaborateurs connus au moment de soumettre le projet.**

   Gayda, Mathieu
   Juneau, Martin
   Lesage, Frederic
   Thorin, Éric
   Bherer, Louis
   Mekary, Saïd
   Renaud, Mélanie
   Lambert, Jean
   Grémeaux, Vincent
   Fraser, Sarah
   Dupuy, Olivier
   Lapierre, Gabriel
   Labelle, Véronique

4. **Demande soumise par :**

   Lalongé, Julie

Renseignements relatifs à la déviation

1. **De quel type de déviation s'agit-il?**

   Déviation mineure

2. **# Participant(e):**

Groupe sujets sains,Groupe sujets MetS, Groupe sujets CHD

Date de la déviation:

2013-12-03

Description de la déviation:

Au protocole institutionnel, à la page 14/31 sous méthodes et mesures à effectuer, à la description de la population, nous avions 20 sujets sains, 20 sujets syndrome métabolique et 20 sujets coronariens à recruter. Nous avons recruté 40 sujets sains, 38 sujets avec syndrome métabolique et 25 sujets coronariens. Une taille d'échantillon plus élevée s'avère nécessaire pour augmenter la puissance statistique.

Commentaire au besoin:

Nous aimerions par ailleurs ajouter 4 autres sujets âgés sains et 12 autres sujets avec syndrome métabolique.

Signature

1. **J'atteste que les renseignements fournis dans le présent formulaire sont exacts.**

Signature

Nom du signataire:

Julie Lalongé

Date de la signature:

2013-12-03

# du poste ou téléphone

374-1480 #259

Le 20 décembre 2013

Docteur Anil Nigam
À l'attention de : Madame Julie Lalongé
Centre ÉPIC
Institut de Cardiologie de Montréal

Projet #2010-154, 1240 – COGNEX
Oxygénation cérébrale, débit cardiaque, performance cognitive et exercice chez les patients présentant un syndrome métabolique, une maladie coronarienne, ou une insuffisance cardiaque.

Cher Docteur Nigam,

Nous vous adressons la décision du Comité d'éthique lors de l'analyse de vos documents soumis à la réunion du 18 décembre 2013 concernant votre projet.

- Formulaire de notification d'une déviation au protocole de recherche susceptible de remettre en cause l'éthicité d'un projet de recherche (F8 - 1673)

<u>Décision du Comité</u> : Approuvé.

Le Comité prend acte de la déviation au protocole rapportée dans ce formulaire.

Le Comité autorise l'augmentation du nombre de participants. Quatre sujets sains et douze autres avec syndrome métabolique seront ajoutés.

La version no. 4, datée du 18 décembre 2013, du formulaire de consentement français, est approuvée.

Le Comité d'éthique de la recherche et du développement des nouvelles technologies de l'Institut de Cardiologie de Montréal (numéro FWA00003235) est désigné par le gouvernement du Québec (MSSS) et adhère aux directives publiées dans *l'ÉPTC 2 - Énoncé de politique des trois conseils : Éthique de la recherche avec des êtres humains* (2010), conformément au *Plan d'action ministériel en éthique de la recherche et en intégrité scientifique* (MSSS 1998). Le Comité adhère aux exigences édictées pour les Comités d'éthique de la recherche selon la Partie C, Titre 5 du *Règlement sur les aliments et drogues* (C.R.C. ch. 870) et agit en conformité avec les standards du *United States Code of Federal Regulations* encadrant la recherche impliquant des sujets humains. Le Comité fonctionne de manière compatible avec les standards internationaux en appliquant notamment la ligne directrice de l'ICH adoptée par Santé Canada : *Les bonnes pratiques cliniques : directives consolidées*.

Notez que la version électronique no. 4 du formulaire de consentement français, datée et approuvée le 18 décembre 2013 est disponible dans Nagano, sous l'onglet Mes Projets/Fichiers/FCE de votre projet de recherche.

Veuillez agréer, Cher Docteur Nigam, l'expression de mes sentiments les meilleurs.

Michel Carrier, MD
Chirurgien cardiovasculaire et thoracique
Président du Comité d'éthique de la recherche et
du développement des nouvelles technologies
MC/fd
